# Multi‐omics analysis of intra‐tumoural and inter‐tumoural heterogeneity in pancreatic ductal adenocarcinoma

**DOI:** 10.1002/ctm2.670

**Published:** 2022-01-21

**Authors:** Xiaoqian Liu, Wenqian Wang, Xiaoding Liu, Zhiwen Zhang, Lianyuan Yu, Ruiyu Li, Dan Guo, Weijing Cai, Xueping Quan, Huanwen Wu, Menghua Dai, Zhiyong Liang

**Affiliations:** ^1^ Department of Pathology State Key Laboratory of Complex Severe and Rare Diseases Molecular Pathology Research Center Peking Union Medical College Hospital Chinese Academy of Medical Sciences and Peking Union Medical College Beijing China; ^2^ Department of Pathology Qilu Hospital (Qingdao) Cheeloo College of Medicine Shandong University Qingdao Shandong China; ^3^ Department of General Surgery Peking Union Medical College Hospital Chinese Academy of Medical Sciences and Peking Union Medical College Beijing China; ^4^ Clinical Biobank Medical Research Centre Peking Union Medical College Hospital Chinese Academy of Medical Sciences and Peking Union Medical College Beijing China; ^5^ Shanghai Tongshu Biotechnology Co., Ltd Shanghai China

**Keywords:** cancer evolution, heterogeneity, immunotherapy, molecular targeted therapy, multi‐omics analysis, pancreatic ductal adenocarcinoma

## Abstract

The poor prognosis of pancreatic ductal adenocarcinoma (PDAC) is associated with the tumour heterogeneity. To explore intra‐ and inter‐tumoural heterogeneity in PDAC, we analysed the multi‐omics profiles of 61 PDAC lesion samples, along with the matched pancreatic normal tissue samples, from 19 PDAC patients. Haematoxylin and Eosin (H&E) staining revealed that diversely differentiated lesions coexisted both within and across individual tumours. Whole exome sequencing (WES) of samples from multi‐region revealed diverse types of mutations in diverse genes between cancer cells within a tumour and between tumours from different individuals. The copy number variation (CNV) analysis also showed that PDAC exhibited intra‐ and inter‐tumoural heterogeneity in CNV and that high average CNV burden was associated poor prognosis of the patients. Phylogenetic tree analysis and clonality/timing analysis of mutations displayed diverse evolutionary pathways and spatiotemporal characteristics of genomic alterations between different lesions from the same or different tumours. Hierarchical clustering analysis illustrated higher inter‐tumoural heterogeneity than intra‐tumoural heterogeneity of PDAC at the transcriptional levels as lesions from the same patients are grouped into a single cluster. Immune marker genes are differentially expressed in different regions and tumour samples as shown by tumour microenvironment (TME) analysis. TME appeared to be more heterogeneous than tumour cells in the same patient. Lesion‐specific differentially methylated regions (DMRs) were identified by methylated DNA immunoprecipitation sequencing (MeDIP‐seq). Furthermore, the integration analysis of multi‐omics data showed that the mRNA levels of some genes, such as *PLCB4*, were significantly correlated with the gene copy numbers. The mRNA expressions of potential PDAC biomarkers *ZNF521* and *KDM6A* were correlated with copy number alteration and methylation, respectively. Taken together, our results provide a comprehensive view of molecular heterogeneity and evolutionary trajectories of PDAC and may guide personalised treatment strategies in PDAC therapy.

## Graphical abstract

Our results provide a comprehensive view of molecular heterogeneity and evolutionary trajectories of PDAC and may guide personalized treatment strategies in PDAC therapy.

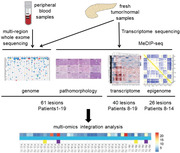

## 1 INTRODUCTION

Pancreatic ductal adenocarcinomas (PDAC) is an aggressive malignancy with an overall 5‐year survival rate of less than 8%.^1^ Many patients develop postoperative recurrence or metastasis, even if diagnosed early and treated timely. Although researchers have made considerable effort to identify biomarkers and therapeutic targets for PDAC using high‐throughput approaches,^2^, ^3^, ^4^ few of them have been applied in clinical practice. Thus, it is urgently needed to develop novel and effective therapeutic strategies for PDAC treatment.

The limited success of conventional therapies for PDAC is partly due to the tumour heterogeneity.^5^ Recent multi‐omics analysis, including genomics, transcriptomics, proteomics and metabolomics, have highlighted the high degree of inter‐tumoural heterogeneity of PDAC between individuals and intra‐tumoural heterogeneity within the same tumour. Both inter‐ and intra‐tumoural heterogeneity may occur at the histological and molecular levels.^6^ At the histological level, different PDAC subtypes classified by the World Health Organization (WHO), including ductal adenocarcinoma, adenosquamous carcinoma, colloid carcinoma and other carcinomas, reflect the histological inter‐tumoural heterogeneity of PDAC.^7^ Different patterns of PDAC, such as conventional ductal adenocarcinomas, intestinal type adenocarcinomas and cystic papillary, may coexist within the same tumour, suggesting histological intra‐tumoural heterogeneity of PDAC.^8^, ^9^ At the molecular level, comparative genomic hybridisation has revealed the differences of genomic abnormalities among multi‐region samples in PDAC.^10^ A study in xenografted mice has shown significant transcriptomic differences between the central and peripheral zones of pancreatic tumours.^11^ Distinct subclonal populations of primary PDAC share identical driver mutations, suggesting that intra‐tumoural heterogeneity might be driven by epigenomic reprogramming.^12^ Studies have also demonstrated that intra‐tumoural heterogeneity represents a snapshot of cancer evolutionary path and may cause treatment failure in clinical practice.^5^, ^13^, ^14^ Although integrated genomics, transcriptomics and epigenomics analyses have provided insights into potential therapeutic strategies for PDAC, there is a lack of data regarding the heterogeneity analysis based on the multi‐omics profiles and tumour evolution of PDAC. Also, the association of tumour heterogeneity with the prognosis of PDAC patients remains unclear.

In this study, by analysing the whole‐exome sequencing (WES), RNA‐sequencing (RNA‐seq) and methylated DNA immunoprecipitation sequencing (MeDIP‐seq) profiles of the paired PDAC and normal pancreatic tissue samples from PDAC patients, we investigated intra‐ and inter‐tumoural heterogeneity at genomic, transcriptomic and epigenomic levels in PDAC. We further integrated multi‐omics data to better understand the complex heterogeneity and identify potential biomarkers for PDAC. Our results provide valuable information about the pathogenesis and prognosis of PDAC.

1 **HIGHLIGHTS**

- Our results provide a comprehensive view of molecular heterogeneity and evolutionary trajectories of PDAC.
- The loss of copy number of *KDM6A* and the low expression of *ZNF521* may be the potential biological indicators of PDAC.
- A higher average CNV burden may be a potential prognostic factor for PDAC.

## 2 RESULTS

### 2.1 Histological heterogeneity analysis

**FIGURE 1:**
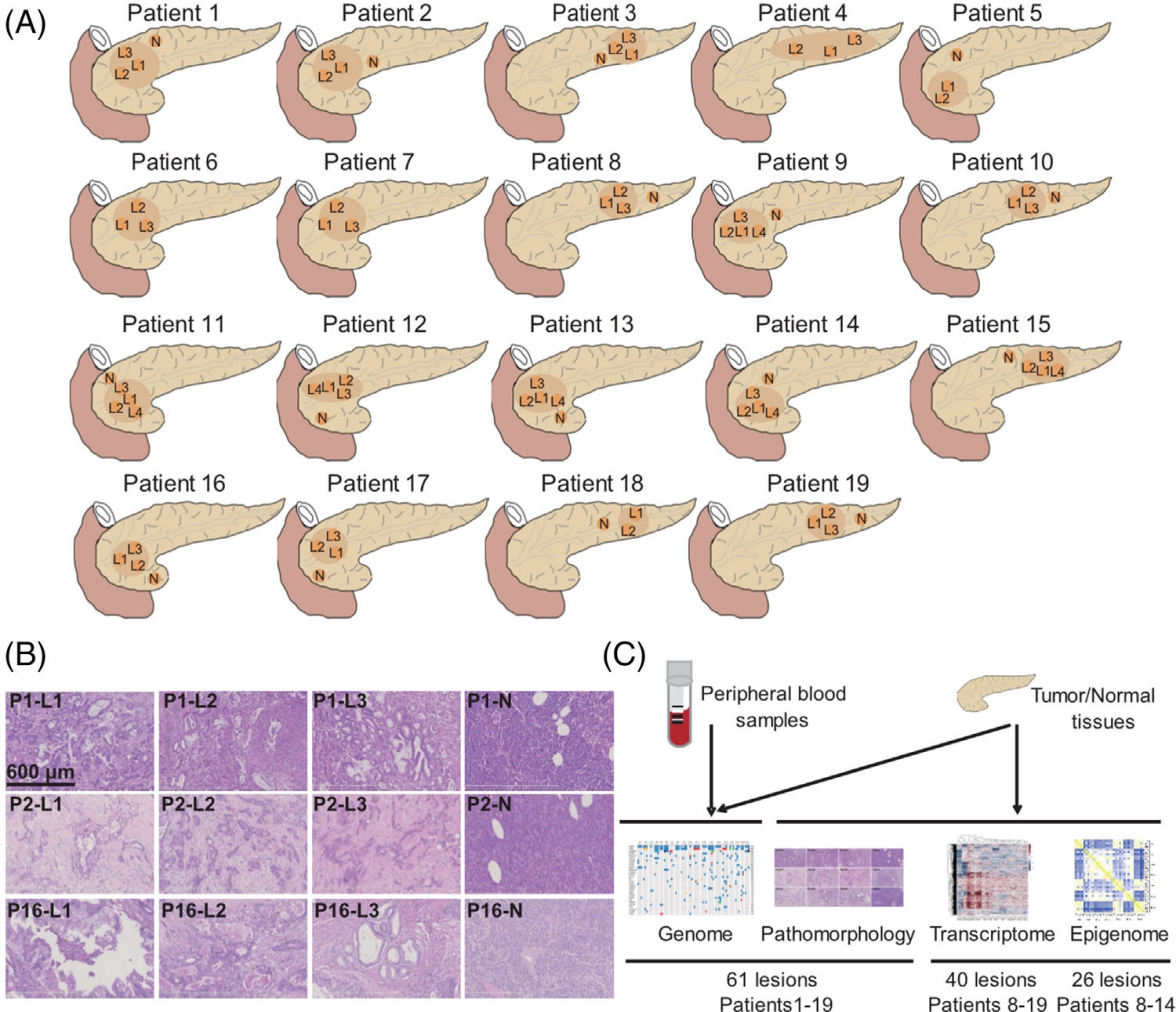
Specimens and workflow. (A) The locations of samples acquired from each patient. A total of 61 lesion samples, together with the matched normal pancreatic tissue samples, were obtained from 19 patients with pancreatic ductal adenocarcinoma (PDAC). (B) The morphological heterogeneity of representative samples. P, patient, L, lesion and N, matched normal tissue. Scale bar = 600 μm. (C) A schematic diagram of multi‐omics analysis

To explore the histological heterogeneity of PDAC, 61 lesions from 19 patients (Figure 1A) were observed under the microscope. Haematoxylin and Eosin (H&E) staining showed that well differentiated (P1–L1, P1–L3, P16–L3) and moderately differentiated lesions (P1–L2, P16–L1, P16–L2) coexisted within the same tumour (Figure 1B), suggesting the histological intra‐tumoural heterogeneity of PDAC. We also observed different differentiation status across individual tumours (supplementary Figure S1), suggesting the histological inter‐tumoural heterogeneity of PDAC.

### 2.2 Mutational and CNV heterogeneity analysis

To explore the heterogeneity of PDAC at the molecular level, we performed multi‐omics analysis to obtain genomic, transcriptomic and epigenomic profiles of the lesion samples following the workflow shown in Figure 1C.

To explore the mutational heterogeneity, we performed multi‐region WES to identify somatic mutations and driver gene mutations in the lesion samples. A total of 12 974 nonsynonymous somatic mutations were identified in 7377 genes, and 5451 synonymous somatic mutations were identified in 3740 genes (supplementary Table S1). Somatic mutations differed between lesions from the same tumours, with missense mutations being dominant mutations (91.8%), followed by nonsense mutations (5.1%) and splice site mutations (2.4%). These data suggest intra‐tumoural heterogeneity of mutation type in PDAC. For example, P6 had multi‐hit *CDKN2A* mutation in L1 and missense *CDKN2A* mutations in L2 and L3.

**FIGURE 2:**
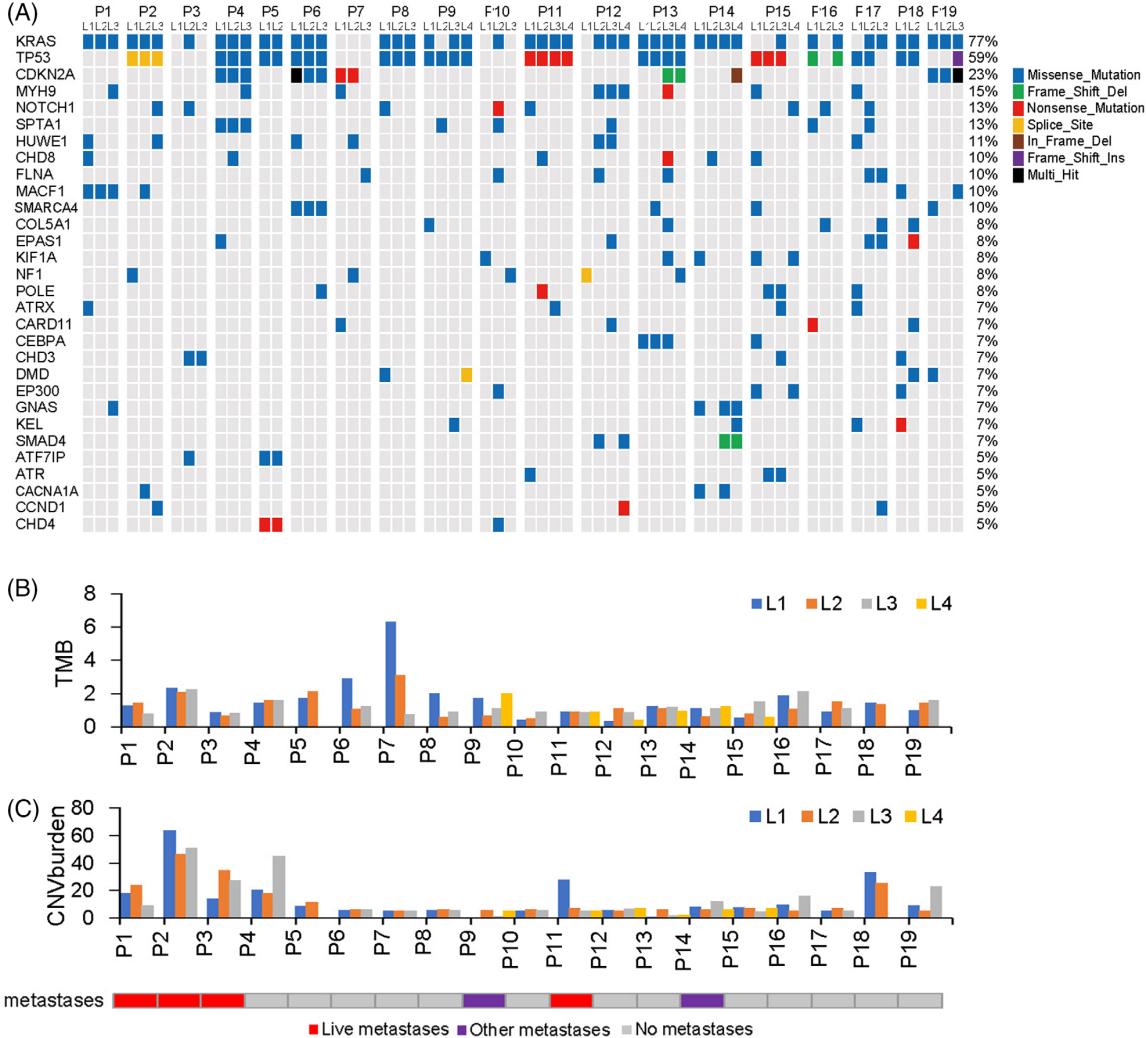
Somatic mutations of driver genes and copy number variations (CNVs) in PDAC. (A) The classification of somatic mutations. (B, C) Comparison of TMB and CNV burden within and across individual tumours. TMB, tumour mutation burden

The most common driver gene mutations occurred in *KRAS* (77.0%), *TP53* (59.0%) and *CDKN2A* (23.0%) (Figure 2A). Mutation frequencies for all the other driver genes were provided in supplementary Table S2.

Regarding the tumour mutational burden (TMB), we found that the mean value of TMB of all the lesions was 1.11 mutations/MB, ranging from 0.37 to 6.29 mutations/MB (Figure 2B). Except for P7–L1 (6.29 mutations/MB), all other lesions showed low TMB values (≤5 mutations/MB). These results also suggest that PDAC patients exhibit inter‐tumoural heterogeneity of TMB.

We further analysed the copy number variation (CNV) heterogeneity. Gain of chromosomes 8q24.22, 16p11.2, 9p12, 9p11.2 and 3q29 and loss of chromosomes 4p13, 21q22.11, 18p11.23, 7q36.3 and 13q12.12 were the most common CNVs (supplementary Figure S2A). Some chromosomes with CNVs carry PDAC driver genes, for example, driver gene *U2AF1* is located in 21q22.3 and *CDKN2A* in 9p24.2 (supplementary Table S3). The degree of CNV, CNV burden and genome instability index (wGII) varied within or across individual tumours (supplementary Figure S2B and S2C and Figure 2C).

### 2.3 Spatiotemporal heterogeneity analysis of genomic alterations

**FIGURE 3:**
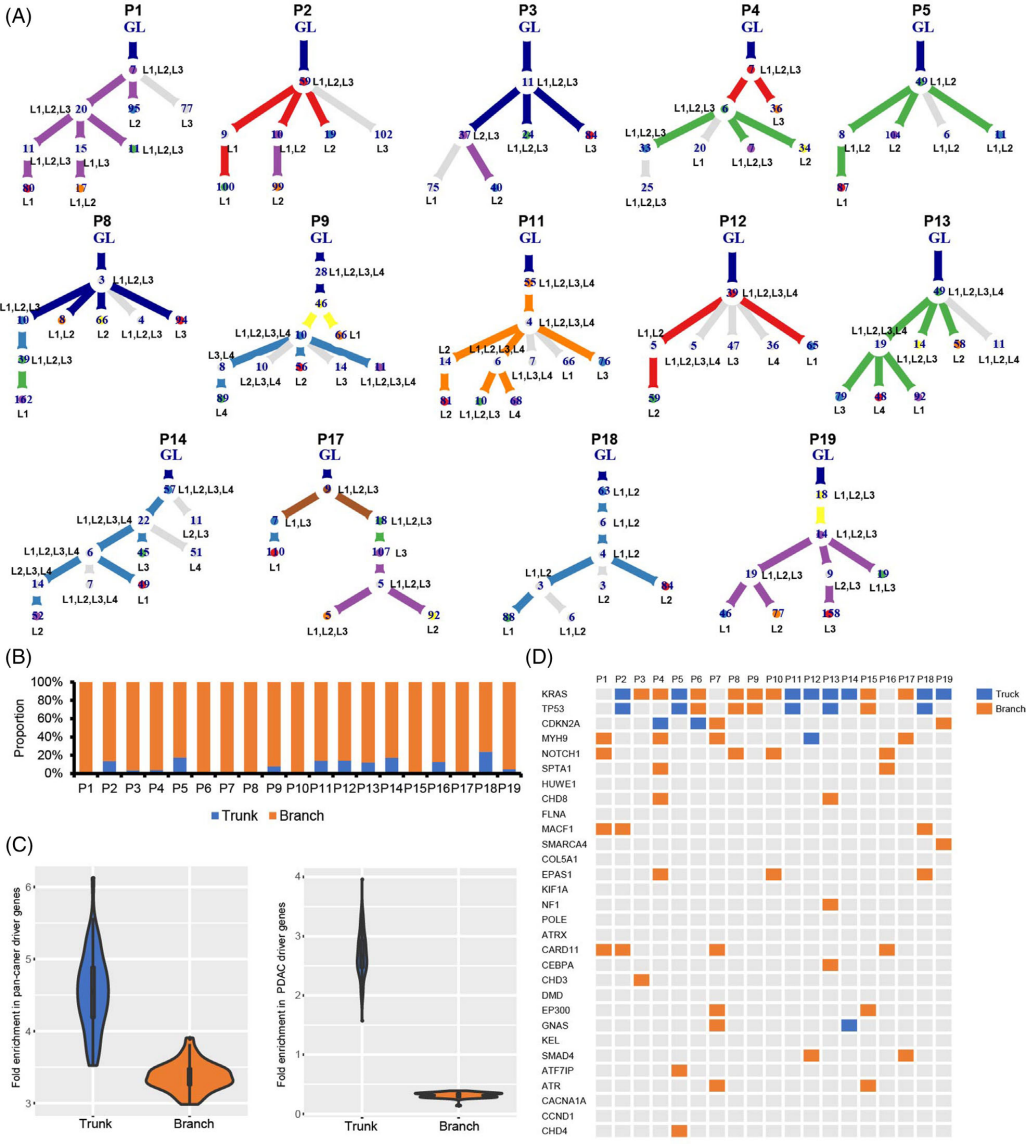
Tumour evolution analysis. (A) Phylogenetic trees were plotted to show the clonal evolution of each sample using Revolver. The grey circle denotes the cluster without driver gene mutations. The coloured circle denotes the cluster with one or more driver gene mutations. The number in the circle represents the quantity of single nucleotide variants. (B) The proportion of trunk/branch mutations in each patient based on phylogenetic trees. (C) The probability density of driver gene fold enrichment among trunk and branch mutations in pan‐cancer and PDAC driver genes. (D) Distribution of most frequently mutated driver genes on phylogenetic trees. GL, germline

To study spatial and temporal heterogeneity of genomic alterations in PDAC, we performed phylogenetic tree analysis. As shown in Figure 3A and supplementary Figure S3, different lesions within or across individual tumours exhibited different evolutionary paths. Each patient had 6–10 clusters in the phylogenetic tree, and the number of SNVs in the trunk varied from 3 to 63. Branch variants (median, 91.9%; range, 76.05%–99.78%) were dominant in all patients compared with trunk variants (Figure 3B). Of note, although SNVs located in the trunk were significantly less than those located in the branch, pan‐cancer (trunk 95% CI: 4.474011–4.704836, branch 95% CI: 3.358584–3.439444) and PDAC driver mutations (trunk 95% CI: 2.619689–2.769267, branch 95% CI: 0.307896–0.3251047) were more likely located in the trunk of the phylogenetic tree (Figure 3C). Specifically, *KRAS*, *TP53* and *CDKN2A* mutations were more likely located in the trunk compared with other driver mutations (Figure 3D).

**FIGURE 4:**
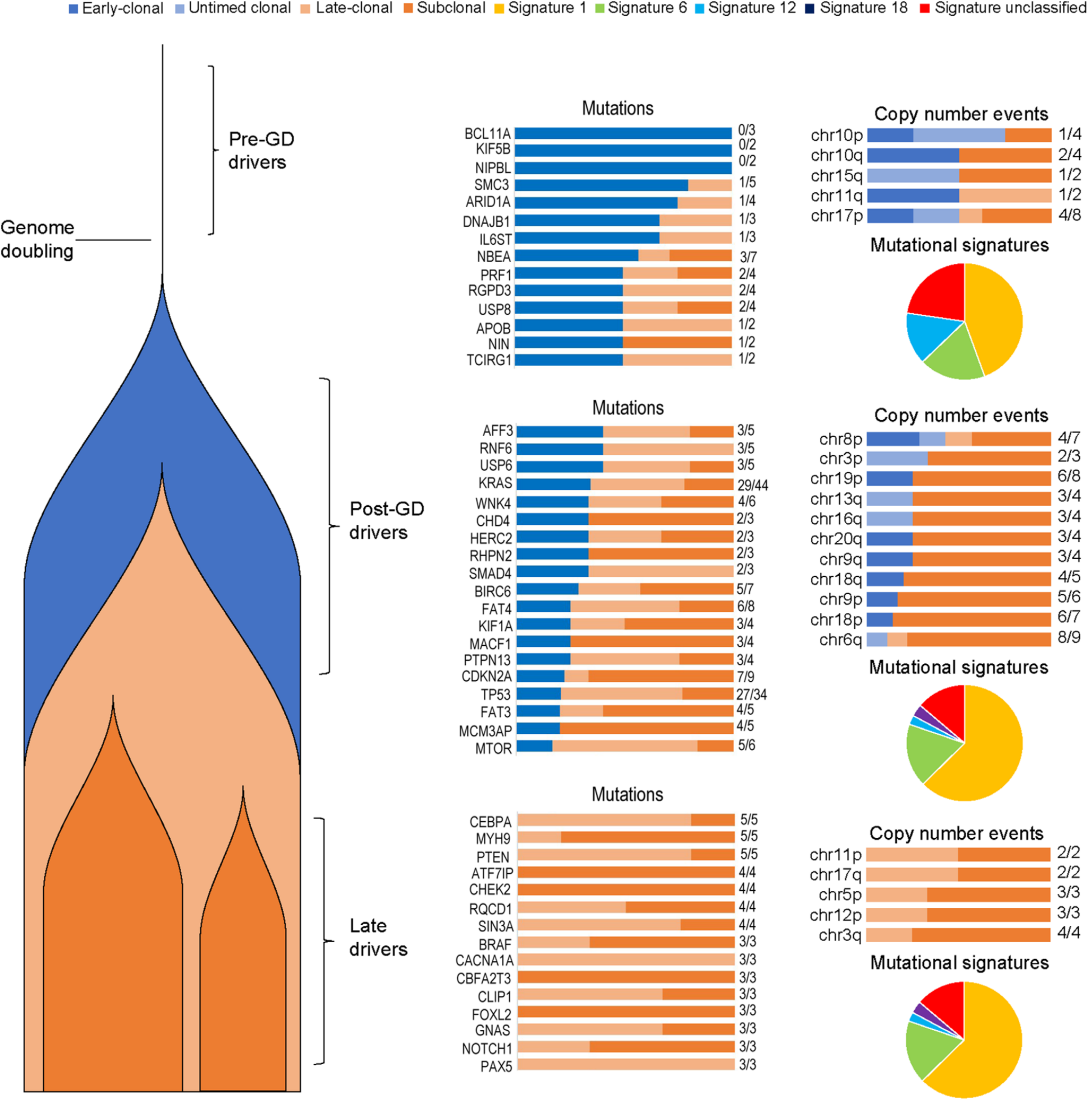
Timing of somatic events in PDAC evolution. The somatic mutations and chromosome‐arms are represented by bars indicating whether the events are clonal or subclonal. Clonal somatic mutations, chromosome‐arms and mutational signatures are further classified as early (before genome duplication) or late (after genome duplication). The frequency of somatic mutations and chromosome‐arm calculated by (late clonal + subclonal)/total are indicated on the right side of the bars. The pie charts show the proportion of each signature. GD, genome doubling

To investigate the temporal heterogeneity of genomic alterations, we evaluated the clonality and timing of somatic mutations, chromosome arms and mutational signature of the lesions. As shown in Figure 4, some alterations, such as *BCL11A* mutation, *KIF5B* mutation and chromosome‐arm 11q loss, were completely or predominantly classified as early‐clonal and occurred before genome duplication, suggesting that these alterations were initial genomic events. Some alterations, such as *CEBPA* and *CACNA1A* mutations, were completely or predominantly late‐clonal and often occurred after genome duplication, suggesting that these alterations contribute to tumour maintenance and progression. Other alterations, such as *SMAD4, KRAS* and *TP53* mutations, were predominantly clonal events in both early and late stages, suggesting that these alterations play important roles across tumour initiation and progression. The mutations in some driver genes, such as *ATF71P*, *CHEK2* and *CBFA2T3*, were classified as subclonal. We also observed temporal intra‐tumoural heterogeneity in *KRAS*, *CDKN2A*, *SMAD4* and *TP53* mutations. For example, the *KRAS* mutations were classified as early‐clonal, late‐clonal and subclonal, in P12–L3, P12–L2 and P12–L4, respectively (supplementary Table S4). Taken together, these data suggest that genomic alterations exhibit spatial and temporal heterogeneities within and across individual tumours in PDAC.

### 2.4 Transcriptional heterogeneity analysis

**FIGURE 5:**
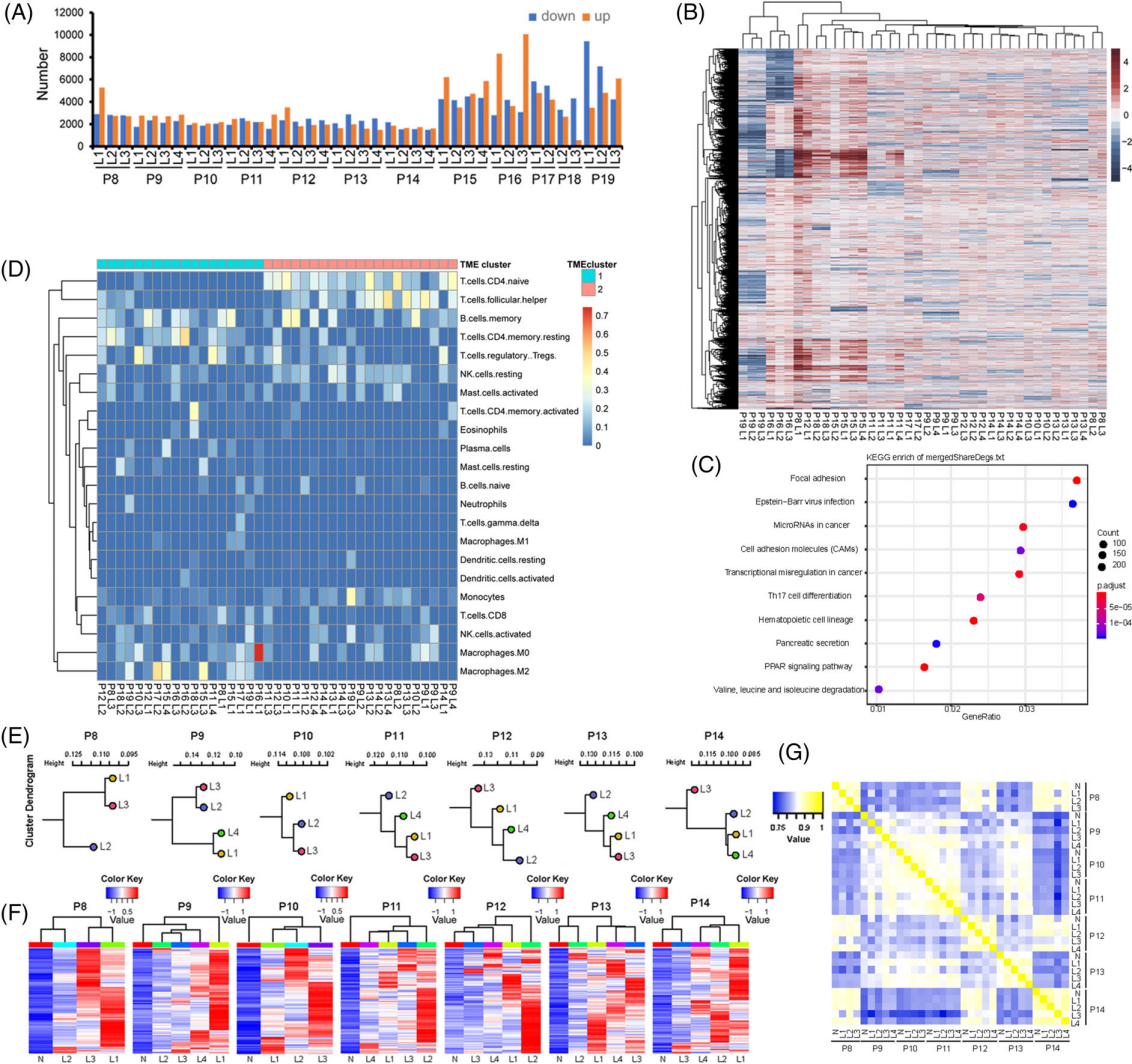
Transcriptional and epigenetic heterogeneities of PDAC. (A) The number of differentially expressed genes (DEGs), which were defined as genes with an absolute GFOLD value greater than 2. The matched normal tissues were used as control. (B) A heatmap of all the downregulated and upregulated DEGs. Red represents upregulated DEG, and blue represents downregulated DEG. (C) KEGG pathway enrichment analysis of the shared DEGs. The DEGs shared by all the lesions within the same tumour were defined as shared DEGs. (D) The heatmap of 22 differentially expressed TME‐related immune markers. Absolute immune cell abundance was calculated using a set of 22 immune cell reference profiles (LM22) on CIBERSORT website to analyse the heterogeneity of the immune microenvironment in each tumour sample. TME, tumour microenvironment. (E) Phyloepigenetic trees. (F) A heatmap of differentially methylated regions in 26 lesions from 7 patients with PDAC. Blue represents hypomethylation, and red represents hypermethylation. (G) Heatmaps show the heterogeneity with the top 2000 hypermethylated regions in the tumour. Yellow represents high similarity. Blue represents low similarity

To evaluate transcriptional heterogeneity of PDAC, we performed RNA‐seq in 40 lesion samples, together with the matched normal tissue samples, from randomly selected 12 PDAC patients. The number of differentially expressed genes (DEGs) varied among the lesion samples within or across individual tumours (Figure 5A), suggesting transcriptional inter‐ and intra‐tumoural heterogeneity in PDAC. Hierarchical clustering analysis was performed on all downregulated and upregulated DEGs from each lesion sample. Samples from the same patients displayed shorter distance between each other than their distances to samples from other patients and are clustered together except P8–L1 and P12–L1 (Figure 5B). We further found that shared DEGs accounted for a small proportion of all DEGs (supplementary Figure S4A), and even the most frequent DEGs were only shared by a maximum of five patients (supplementary Table S5). In addition, PCA cluster analysis was also performed using the fold‐change of gene expression as input, and the results showed that samples from each individual tend to form a single cluster (supplementary Figure S5A). These data indicated that PDAC exhibits a lower degree of intra‐tumoural heterogeneity than inter‐tumoural heterogeneity at the transcriptional level. Kyoto Encyclopaedia of Genes and Genomes (KEGG) pathway enrichment analysis of DEGs showed differences among the lesions within the same tumour, suggesting an intra‐tumoural heterogeneity of the signalling pathways (supplementary Figure S6A). The shared DEGs were enriched in important tumour onset and metastasis pathways, such as focal adhesion, cell adhesion molecules and transcriptional mis‐regulation in cancer endoplasmic reticulum (Figure 5C).

### 2.5 PDAC microenvironmental heterogeneity analysis

Samples with tumour microenvironment (TME) expression profiles were divided into two clusters by unsupervised clustering analysis using CIBERSORT, an analytical tool from the Alizadeh Lab developed by Newman et al. using gene expression data to provide an estimation of the abundances of member cell types in a mixed cell population^15^ (Figure 5D). The two clusters presented different immune cell profiles. Briefly, the proportions of CD4 naive T cells and T follicular helper cells were significantly higher in cluster 2 than those in cluster 1 (*p* = 1.9^e‐9^ and *p* = 4.9^e‐4^, respectively). This finding was further verified by immunohistochemical (IHC) and multiplex immunofluorescence (IF) staining (supplementary Figure S5B–D). The results showed that the cluster 2 samples were characterised by higher density or proportion of T follicular helper cells using IHC (*p* = .0253) and multiplex IF (*p* = .005) staining, respectively. TME clustering showed the differential expression of immune markers within and across individual tumours. Lesions from the same tumour did not cluster together, suggesting that TME is more heterogeneous than tumour cells in the same patient.

### 2.6 DNA methylation heterogeneity analysis

Considering that genomic and transcriptomic heterogeneity are insufficient to explain the phenotypic diversity of tumours, we performed MeDIP‐seq to investigate the heterogeneity of PDAC at the epigenetic level. Figure 5E illustrates the phyloepigenetic trees of seven randomly selected PDAC patients. We found that differentially methylated regions (DMRs) differ substantially between lesions from the same patients, reflecting intra‐tumoural heterogeneity of PDAC (Figure 5F). These DMRs were further divided into shared DMRs and private DMRs. The number of shared DMRs (median, 11 193; range, 546–20 736) was less than that of private DMRs (median, 167 910; range, 95 907–219 063) (supplementary Figure S4B). Analysis of the hypermethylated areas in the tumour further demonstrated intra‐tumoural heterogeneity, and intra‐tumoural heterogeneity was lower than that of inter‐tumoural heterogeneity (Figure 5G). KEGG analysis showed that the genes with shared DMRs were enriched in cancer‐related pathways, such as Receptor Tyrosine Kinases, Rho GTPases and Neuronal System (supplementary Figure S6B). However, the shared DMRs were mainly located in introns, and no invariably‐hypermethylated promoters were enriched in the KEGG pathway. Taken together, these data suggest that PDAC exhibits intra‐ and inter‐tumoural heterogeneity at the epigenetic level.

### 2.7 Multi‐omics integration analysis

**FIGURE 6:**
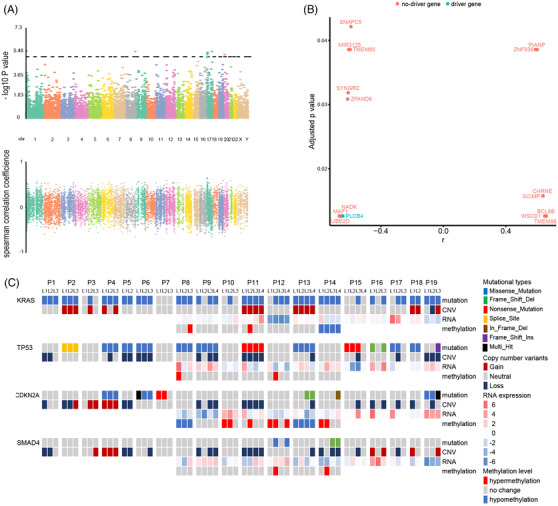
The association of genomic alterations with RNA expression. (A) Correlation between CNV and mRNA expression in all samples. (B) The RNA expressions of some genes were significantly correlated with the gene copy numbers. Blue dot represents driver gene, and red dot represents non‐driver gene. (C) A heatmap of mutations, CNV and their associations with RNA expression of the most frequently mutated PDAC driver genes

To better understand the complex heterogeneity in PDAC, we integrated multiple omics data, including the mutation types, copy number alterations, mRNA expression and methylation status. As shown in Figure 6A and B, the mRNA levels of some genes were significantly correlated with the copy numbers, including PDAC driver gene *PLCB4*. Figure 6C shows the integration of multi‐omics data within and across individual tumours. We observed that, of the four lesions of P14, the mutation status, CNV and methylation of *KRAS* remained unchanged, whereas the mRNA expression of *KRAS* was different. The same trend was observed in *TP53* within the tumour of P11. These results suggest that, in addition to mutation status, CNV and DNA methylation, other factors may affect PDAC driver gene expression.

**FIGURE 7:**
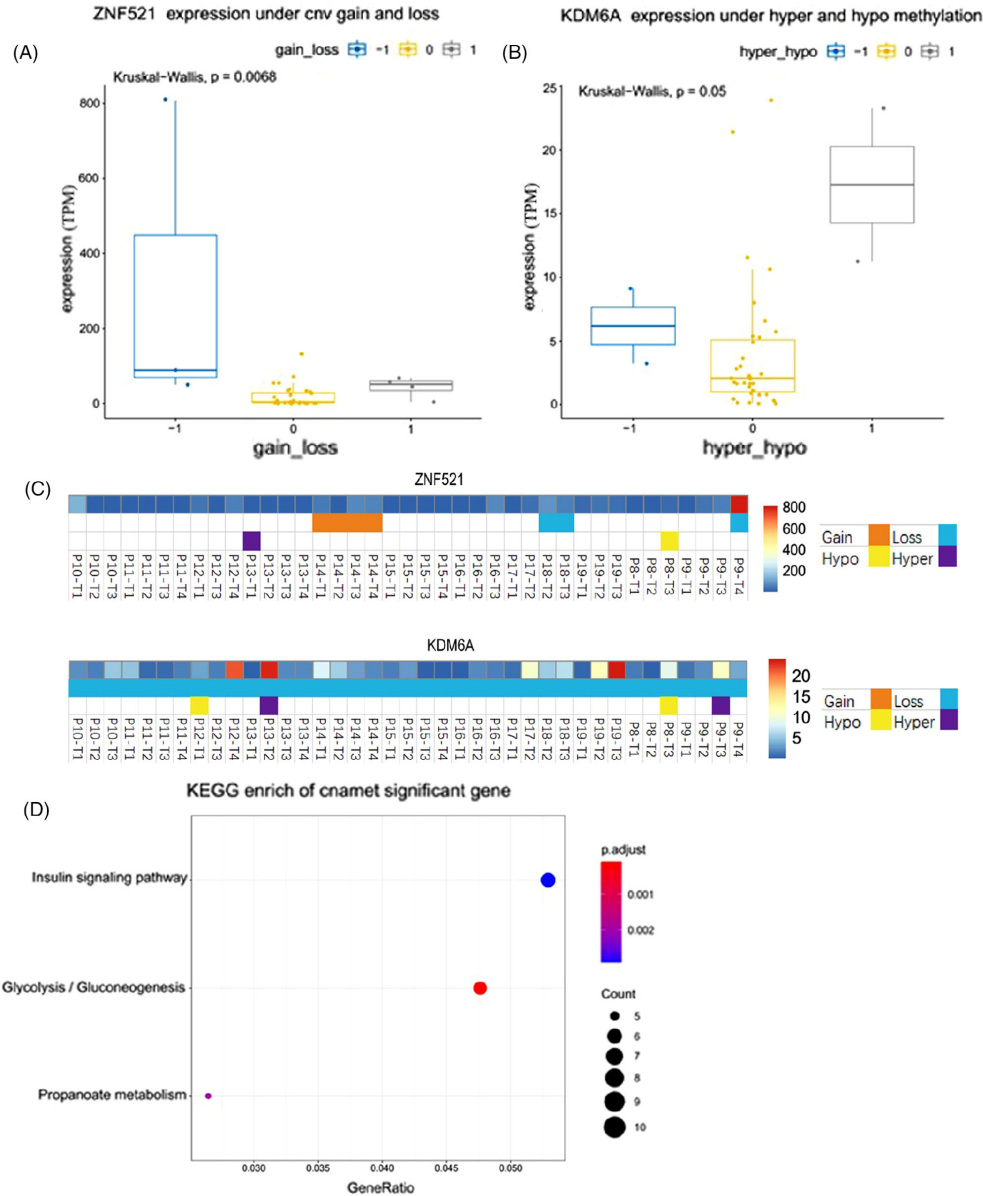
Correlation of mRNA expression of some genes with CNV or methylation status. (A) Correlation between mRNA expression and methylation status of ZNF521. (B) Correlation between mRNA expression and CNV of KDM6A. (C) The distribution of KDM6A and ZNF521 expression, copy number and methylation status across samples. (D) KEGG pathway enrichment analysis of key genes. Hyper/hypo represents the degree of methylation. –1 and 1 represent hypomethylated and hypermethylated states, respectively. 0 indicates no obvious change in methylation state. Gain/loss represents the change of copy number, –1 and 1 represent the deletion or amplification of copy number respectively, and 0 represents the insignificant change of copy number

We further found that *ZNF521* expression was positively correlated with copy number alterations (*p *= .0068; Figure 7A) and that *KDM6A* expression was negatively correlated with DNA methylation (*p* = .05; Figure 7B). *ZNF521* has consistently lower expression in all the samples from tumours P8–P19, except P9–T4 (Figure 7C, upper panel). Similarly, the loss of *KDM6A* copy number was also detected in all samples (Figure 7C, lower panel), despite *KDM6A*’s diverse expression and methylation status within and across individual tumours from P8 to P19. These findings suggested that low expression of *ZNF521* and the loss of *KDM6A* copy number may work as biomarkers for PDAC.

In addition to *ZNF521* and *KDM6A*, we performed a systematic analysis to identify non‐varying genes at the three omics levels through the same approach. Significant correlations between the mRNA expressions and copy number alterations of *MDM4*, *RRAGC*, *HERC2*, *BIRC3* and *TSC2* were observed as well (supplementary Figure S7A). KEGG analysis showed that these genes (supplementary Table S6) were enriched in metabolism‐related pathways such as ‘insulin signalling pathway’, ‘glycolysis/glucogeogenesis’ and ‘propanoate metabolism’ signalling pathways (Figure 7D). However, the gene‐level integrated analysis revealed heterogeneity in expression, copy number variation and methylation status of *MDM4*, *RRAGC*, *HERC2*, *BIRC3* and *TSC2* genes within and across individual tumours (supplementary Figure S7B), suggesting that they may not be ideal biomarkers for PDAC.

ActivePathways^16^ was utilised to identify significantly altered pathways from multiple‐omics data. All genes identified in RNAseq, WES or MEDIP data analyses (supplementary Table S7) were used as inputs for the integrative analysis. The results highlighted 43 genes that were significantly enriched in 13 KEGG pathways. Based on the KEGG database annotation, 10 out of the 13 pathways were found to be frequently enriched in multiple types of cancer. The other three enriched pathways were TP53, cytokine–cytokine receptor interaction and GAP Junction signalling pathway. ActivePathways also identified 26 significantly enriched GO biological processes; 17 of them were supported by all the three types of data (mutation, expression and methylation) (supplementary Figure S8).

### 2.8 Correlation analysis of prognosis in PDAC

**FIGURE 8:**
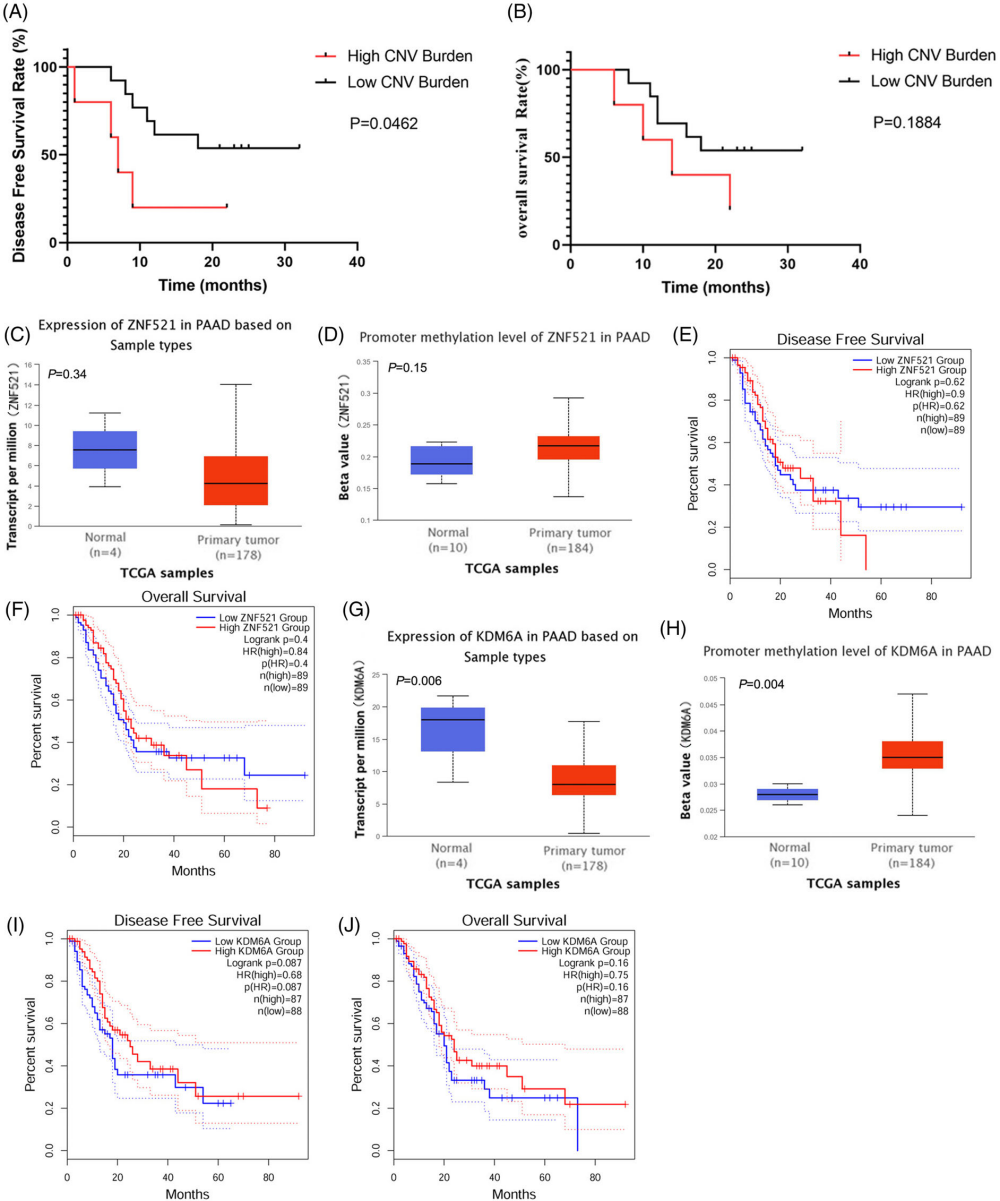
Correlations of CNV burden with patients’ survival and characterisation of KDM6A and ZNF521 in PDAC patients from the The Cancer Genome Atlas (TCGA) database. (A, B) Kaplan–Meier survival curves of patients with low or high average CNV burden. Patients with average CNV burden in the top quartile and the rest were segregated into high and low CNV burden groups, respectively. Disease‐free survival (DFS) was defined as the time from surgery to locoregional recurrence or distant metastasis. Overall survival (OS) was defined as the time from surgery to death from any cause or last follow‐up (censored patient). (C, D) mRNA expression and promoter methylation of ZNF521 in normal tissue and primary tumour tissue from TCGA database. (E and F) Correlations of ZNF521 mRNA expression with disease‐free survival and overall survival of patients from TCGA database. (G, H) mRNA expression and promoter methylation of KDM6A in normal tissue and primary tumour tissue from TCGA database. (I, J) Correlations of KDM6A mRNA expression with disease‐free survival and overall survival of patients from TCGA database. The dotted lines in E, F, I and J represent the error bars of 95% CI. PAAD, pancreatic adenocarcinoma

With the median 14.4 months post‐surgery follow‐up, 6/19 (31.6%) patients were found to have disease relapse and/or metastasis. Liver metastases occurred in 4 patients (P1–3, P11), and disease‐specific death occurred in 10/19 (52.6%) patients (P1–4, P6, P9, P11 and P13‐P15). The patients with liver metastases had significantly higher average CNV burden than other patients (21.12 vs. 6.17, *p* = .033; Table 1). Patients were further stratified by CNV burden quartiles for survival analysis. Patients with CNV burden in the top quartile had significantly shorter survival compared to patients in the other three quartiles [disease‐free survival (DFS), *p* = .0462 and overall survival (OS), *p *= .1884; Figure 8A and B].

**TABLE 1:** Associations between clinicopathological parameters and average CNV burden in 19 PDAC patients

	Average CNV burden *n* (%)		
Parameter	**Low**	**High**	*p* Value
**Total**	10 (52.6)	9 (47.4)	
**Age(y/o)**			.303
≥60	9 (47.4)	6 (31.6)	
<60	1 (5.3)	3 (15.8)	
**Gender**			.999
Male	5 (26.3)	4 (21.1)	
Female	5 (26.3)	5 (26.3)	
**Tumour location**			.650
Head	7 (36.8)	5 (26.3)	
Body/Tail	3 (15.8)	4 (21.1)	
**Tumour size**			.656
>3 cm	6 (31.6)	4 (21.1)	
≤3 cm	4 (21.1)	5 (26.3)	
**Lymph node metastasis**			.656
Yes	4 (21.1)	5 (26.3)	
No	6 (31.6)	4 (21.1)	
**Disease relapse and/or metastasis**			.350
Yes	2 (10.5)	4 (21.1)	
No	8 (42.1)	5 (26.3)	
**Liver metastasis**			.033*
Yes	0 (0.0)	4 (21.1)	
No	10 (52.6)	5 (26.3)	
**Clinical staging**			.999
I	2 (10.5)	1 (5.3)	
II	8 (42.1)	8 (42.1)	

* Means statistically significant.

Data of patients with pancreatic adenocarcinoma from The Cancer Genome Atlas (TCGA) database were analysed as well for comparison. The results of TCGA database showed that the expression of *ZNF521* was decreased in 178 primary tumours compared with 4 normal tissue samples (Figure 8C), and its promoter methylation levels were increased in 184 primary tumours compared with 10 normal tissue samples (Figure 8D), which supported the results of our cohort study that *ZNF521* was under‐expressed in 39 tumour samples from 12 patients. However, no significant correlation was observed between *ZNF521* expression and prognosis of patients (Figure 8E and F). Similar results were observed in the transcription and promoter methylation of *KDM6A* (Figure 8G and H), which showed *KDM6A* expression was not significantly correlated with patient prognosis (Figure 8I and J). We further analysed the association of *ZNF521* or *KDM6A* expression with the prognosis of the patients in our cohort. We found that although patients with low *ZNF521* expression tended to show poor prognosis (supplementary Figure S9), the results were not statistically significant.

## 3 DISCUSSION

Studies on PDAC heterogeneity have mainly focused on WES data,^17^ ^–^ ^19^ and a multi‐omics analysis is lacking. In this study, we evaluated intra‐ and inter‐tumoural heterogeneity of PDAC across genomic, transcriptomic and epigenomic levels and depicted the evolutionary trajectories of the tumours to unveil the spatial and temporal occurrence of genomic alterations. We observed intra‐ and inter‐tumoural heterogeneity in tumour cells and TME of PDAC. The integrated multi‐omics analysis revealed that despite the heterogeneities observed in certain omics, low expression of *ZNF521* and loss of *KDM6A* copy number were consistently presented within and across individual tumours, serving as potential biomarkers for PDAC. In addition, high average CNV burden may be correlated with poor PDAC prognosis, which can be used as a potential prognostic marker for PDAC.

Although the degree of intra‐tumoural heterogeneity was less than that of inter‐tumoural heterogeneity, the distinct genomic profiles of different lesions within a single tumour highlighted the presence of intra‐tumoural heterogeneity in PDAC. Consistent with that reported by Makohon‐Moore et al.,^17^ our phylogenetic tree analysis demonstrated that branch variants were dominant in all lesions, whereas trunk variants were enriched in driver genes of pan‐cancer and PDAC, suggesting that intra‐tumoural genomic heterogeneity is more likely caused by passenger gene mutations. Although it is more likely that intra‐tumoural heterogeneity is contributed to passenger gene mutations, driver gene mutation is also remarkable in PDAC's heterogeneity. Driver gene mutations, which confer selective growth advantages to cancer cells, are critical in tumour evolution, while passenger mutations tend to be random mutations accumulated in cells with no serious functional consequences.^20^ To better understand the role of driver gene mutations in the occurrence and progression of PDAC, we explored the spatial and temporal heterogeneities of PDAC driver gene mutations. In a considerable proportion of the patients, the same driver genes, including *KRAS*, *TP53, SMAD4* and *CDKN2A*, have diverse genotypes and mutation types between lesions from one individual tumour. This suggests the intra‐tumoural spatial heterogeneity of driver gene mutations. Although the clonal status of driver gene mutations has drawn the attention of PDAC researches, little is known about the clonal/subclonal frequency and the timing of mutation during tumour evolution.^21^ In this study, temporal clonal clustering classified driver gene mutations into four types: type 1 mutations were completely or predominantly clonal and occurred before genome duplication, such as *BCL11A, KIF5B* and *NIPBL* mutations; type 2 mutations were completely or predominantly clonal and occurred after genome duplication; type 3 mutations were predominantly clonal events in both early and late stages, including *KRAS, SMAD4* and *TP53* mutations; type 4 mutations were classified as subclonal. Mutations of the three major PDAC driver genes *KRAS*, *TP53* and *SMAD4* ^22^ were predominantly clonal events, consistent with their critical roles in PDAC. In terms of the timing, they might participate in both tumour initiation and progression, suggesting the differential roles of these genes during tumour evolution. The occurrence of subclonal *KRAS* mutations has been reported in PDAC,^23^ supporting the temporal intra‐tumoural heterogeneity of PDAC driver gene mutations observed in this study. The mutations in other driver genes were mainly late and subclonal events, demonstrating a significant intra‐tumoural heterogeneity. The spatial and temporal intra‐tumoural heterogeneity of driver gene mutations may contribute to therapeutic resistance in PDAC, leading to treatment failure.

In addition, following the classification criteria of TMB levels in a large cohort study which approximately divided ∼50% of patients to low TMB (1–5 mutations/MB), ∼40% intermediate TMB (6–19 mutations/MB) and 10% high TMB (≥20 mutations/MB),^24^ almost all the lesions in our study showed low TMB, consistent with the results of other large PDAC cohort studies.^22^, ^25^

In our study, although the degree of inter‐tumoural heterogeneity was higher than that of intra‐tumoural heterogeneity at the transcriptional level, the presence of intra‐tumoural transcriptional heterogeneity was unignorable. Importantly, we observed that the PDAC TME was relatively more heterogeneous than tumour cells, possibly due to the complicated components in the PDAC TME. The PDAC TME is characterised by abundant fibrotic stroma (desmoplasia) that includes a heterogeneous mixture of pancreatic stellate cells (PSCs), immune cells and extracellular matrix (ECM). Our previous study had demonstrated that PSCs and ECM promote cell proliferation, invasion, migration and drug resistance of PDAC cell lines, suggesting an application of targeted strategies tailored to the PDAC TME.^26^, ^27^, ^28^, ^29^, ^30^ In this study, hierarchical clustering analysis of immune cells in TME revealed all tumour samples could be clustered into two clusters, which was further verified by IHC and multiplex IF staining, highlighting the importance and potential of targeted therapy and immunotherapy in PDAC.

We further explored potential prognostic factors for PDAC by integrating the multi‐omics data, including copy number alterations, mRNA expression and methylation status, and analysing their associations with the prognosis of patients with PDAC. Studies have shown that CNV burden is a potential prognostic biomarker for human cancers.^31^, ^32^ However, the prognostic value of CNV in PDAC remains unknown. Our results showed that the patients with liver metastases had significantly higher average CNV burden than other patients. Moreover, patients with high average CNV burden had significantly shorter DFS than those with low average CNV burden. These results suggest that the CNV burden is associated with the prognosis of the patients, serving as a potential prognostic factor for PDAC.

Murphy et al have demonstrated that *ZNF521* is one of the most frequently mutated genes during PDAC progression. *ZNF521* exhibits chromosomal rearrangement or loss in 63% PDAC tumours and an overall trend of reduced expression in PDAC tumours compared with matched normal ductal epithelial cells.^33^ Consistently, we found that *ZNF521* was lowly expressed in all the samples from tumours P8–P19, except P9–T4, suggesting that low expression of *ZNF521* may serve as a biomarker for PDAC. Although the high expression level of *ZNF521* in P9–T4 seems to be unusual, this finding is perhaps not surprising given the relatively high intra‐tumoural heterogeneity at the expression level in our PDAC cohort. It indicates the complexity of intra‐tumoural heterogeneity and suggests the necessity of precision/personalised medicine strategies to improve patients' outcomes. Similarly, several previous studies reported highly discordant gene expression in different areas within individual tumours due to heterogeneity.^34^, ^35^, ^36^, ^37^

Each gene's copy number was calculated in this study based on the mapped bam file of NGS reads for each sample. Since we had no access to the raw data or bam files of samples in TCGA, it would take several months to go through the application process even if the application was finally granted. Alternatively, we extracted the related information from published literatures using TCGA data. Our results showed that *KDM6A* expression was positively correlated with methylation. We also noticed that although *KDM6A* mRNA expression and methylation exhibited heterogeneities within and across individual tumours, the loss of *KDM6A* copy number remained consistent. This finding suggests that the loss of *KDM6A* copy number may serve as a biomarker for PDAC. This conclusion was supported by the study of Watanabe et al., showing that loss of *KDM6A* in tumour tissue is an independent prognostic factor for recurrence‐free survival and OS of patients with PDAC.^38^ However, we did not observe statistically significant correlation of *ZNF521* or *KDM6A* expression with the prognosis of PDAC patients from our study or TCGA database, possibly due to the small sample size, race or the heterogeneities of *ZNF521* and *KDM6A* expression in PDAC. Therefore, the prognostic value of these genes needs to be further evaluated with a larger sample size.

This study has several limitations. First, it is a retrospective study that there might be some bias in the selection of cases and deficiency in some important medical information of patients. Second, as a retrospective study, we only collected specimens and clinical information for molecular testing and data analysis. No intervention or treatment change was applied to the participants. Next, in prospective studies, the treatment regimens may be decided or optimised on the basis of multi‐omics analysis to achive better outcomes. Third, the patient number of 19 appeared scarce. In this study, we collected both tumour and the corresponding paracancerous tissue samples from 19 PDAC patients to explore the intra‐tumoural and inter‐tumoural heterogeneity of PDAC at three omics (i.e. genomic, transcriptomic and epigenomic) levels as well as evolutionary trajectories. To capture the intra‐tumoural heterogeneity, tumour tissues were sampled at multiple sites from each sample. In addition, matched normal tissue was collected >2 cm from the visible edge of the tumour and did not contain any tumour cells by histopathologic review. It took us 1 year and 4 months (from March 2018 to July 2019) to collect these 19 samples (a total of 80 samples including 61 tumour lesions and 19 matched normal samples) qualified for our analyses, and it is difficult for us to collect more during a limited period. Our data were from the multi‐omics analysis of the resected pancreatic cancer tissues instead of from the existed databases. To our knowledge, our study is the largest cohort to date utilising private muti‐omics data from multi‐regional sampling for heterogeneity analysis in PDAC. Currently, in studies involving multi‐omics (genomic, transcriptomic and epigenomic) analysis and large pancreatic patient number (e.g. over 100), the data were from the TCGA and GEO databases.^39^, ^40^, ^41^ For example, Kong et al. used TCGA and GEO databases to perform genomics, epigenomics and transcriptomics analysis in 161 pancratic cancer patients to identify molecular subgroups and explore novel biomarkers.^39^ Mishra et al. combined multi‐omics data (including gene expression, DNA methylation and miRNA expression data) and survival data of 153 PDAC patients from the TCGA database to identify potential prognostic markers of PDAC.^40^ Given the difference in the genetic characteristics, disease subtypes and status, etc. between the Chinese patient populations and the analysed patients in the existed databases (TCGA, GEO), the data from the databases might not accurately reflect the molecular features of the Chinese PDAC patient population. Therefore, in this study, instead of reviewing the existed database data, we collected the tumour and paracancerous samples from our PDAC cases to systematically explore the intra‐tumoural and inter‐tumoural heterogeneity using multi‐omics sequencing approaches. Further studies with more patients and longer follow‐up period will be carried out to validate this study. Fourth, our study lacks proteomics and single‐cell analyses that may provide more information about heterogeneity, which will be addressed in future study.

## 4 CONCLUSIONS

In conclusion, our multi‐omics analysis provides new insights into the heterogeneity and tumour evolution of PDAC at the molecular level. We also identified high average CNV burden as a potential prognostic factor for PDAC. These findings may facilitate clinical decision making in PDAC therapy.

## 5 MATERIALS AND METHODS

### 5.1 Patients and sample collection

This study was approved by the Ethics Committee of Peking Union Medical College Hospital (S‐K1036). A total of 19 PDAC patients (P1–19; 44–72 years old) who have undergone pancreatic resection from March 2018 to July 2019 were recruited in this study, including 9 males and 10 females. All patients provided written informed consents. The clinical characteristics of the patients were summarised in Table 2. The diagnosis of PDAC was confirmed by two experienced pathologists. The histological grade of PDAC was classified as well, moderately and poorly differentiated according to the WHO Classification of Tumours of the Digestive System.^42^ Well differentiated PDAC are composed of haphazardly arranged in filtrating duct‐like structures and medium‐sized glands. Moderately differentiated PDAC are characterised by abundant glands forming cribriform, papillary, micropapillary and/or gyriform patterns. Foci of smaller and more irregular glands and some individual pleomorphic cells are often found at the tumour margins. And poorly differentiated PDAC are composed of solid or cribriform cell sheets and individual pleomorphic cells embedded in loosely arranged stroma. None of the patients have received chemotherapy or radiotherapy for PDAC. For each patient, the tumour tissue samples were collected during the surgical resection from 2–4 lesions (L1–4) that were at least 0.5 cm away from each other. A total of 61 lesion samples were obtained from 19 patients. The matched normal pancreatic tissue samples and peripheral blood leukocytes were also collected. Matched normal tissue was collected > 2 cm from the visible edge of the tumour and did not contain any tumour cells by histopathologic review. Tissue samples were minced on ice and digested as previously described.^43^ Dissociated cells were washed with phosphate‐buffered saline containing 0.04% bovine serum albumin (Sigma‐Aldrich, St. Louis, MO, USA) before RNA/DNA extraction.

**TABLE 2:** The clinical characteristics of the patients

**Patient ID**	**Gender**	**Age (y/o)**	**status**	**Metastasis after surgery**	**OS (m)**	**DFS (m)**	**Average TMB**	**Average CNV burden**	**Smoking**	**Drinking**	**Hypertension**	**Diabetes mellitus**	**Location**	**Degree of differentiation**	**TNM classification**	**Clinical staging**	**Maximum diameter (cm)**
P1	Female	65	Dead	Yes (liver metastasis)	22	9	1.167	16.927	No	No	Yes	Yes	Head	Well/moderate	T2NXM0	IB	>3
P2	Male	64	Dead	Yes (liver metastasis)	14	7	2.219	53.723	Yes	Yes	Yes	No	Head	Moderate/poor	T3N0M0	IIA	>3
P3	Male	55	Dead	Yes (liver metastasis)	10	1	0.789	25.313	Yes	Yes	Yes	Yes	Body and tail	Moderate	T3N0M0	IIA	≦3
P4	Male	51	Dead	No	6	6	1.535	27.662	Yes	No	No	No	Body and tail	Moderate/poor	T3N1M0	IIB	>3
P5	Female	63	Live	No	32	32	1.921	10.116	No	No	No	No	Head	Well	T2N1Mx	IIB	≦3
P6	Female	64	Dead	No	11	11	1.737	5.742	No	No	No	Yes	Head	Moderate/poor	T3N1M0	IIB	>3
P7	Female	69	Live	No	32	32	3.368	5.234	No	No	Yes	Yes	Head	Moderate	T2N0M0	IB	≦3
P8	Male	44	Live	No	25	25	1.175	5.868	No	Yes	No	No	Body and tail	Moderate	T2N0M0	IB	>3
P9	Female	64	Dead	Yes (peritoneal metastasis)	12	12	1.388	3.242	No	No	Yes	No	Head	Moderate/poor	T3N1M0	IIB	>3
P10	Female	64	Live	No	25	25	0.614	5.686	No	No	Yes	No	Body and tail	Moderate	T3N0M0	IIA	≦3
P11	Female	65	Dead	Yes (liver metastasis)	16	9	0.895	11.446	Yes	No	Yes	No	Head	Moderate/poor	T3N1M0	IIB	≦3
P12	Male	64	Live	No	24	24	0.704	6.175	Yes	Yes	Yes	Yes	Head	Moderate	T3N0M0	IIA	>3
P13	Female	64	Dead	No	8	8	1.125	2.786	No	No	Yes	No	Head	Moderate	T3N1M0	IIB	>3
P14	Male	63	Dead	Yes (pulmonary metastasis)	12	6	1.020	7.993	Yes	No	Yes	Yes	Head	Well/moderate	T3N0M0	IIA	≦3
P15	Male	69	Dead	No	18	18	0.855	6.617	Yes	Yes	Yes	No	Body and tail	Moderate	T3N0M0	IIA	>3
P16	Female	73	Live	No	23	23	1.702	10.246	No	No	No	Yes	Head	Moderate	T3N1M0	IIB	≦3
P17	Male	72	Dead	No	N/A*	N/A*	1.175	5.883	No	No	Yes	Yes	Head	Moderate/poor	T3N1M0	IIB	≦3
P18	Female	64	Live	No	22	22	1.395	29.395	No	No	Yes	No	Body and tail	Moderate/poor	T3N0M0	IIA	≦3
P19	Male	42	Live	No	21	21	1.333	12.493	No	No	No	No	Body and tail	Moderate	T3N1M0	IIB	>3

* P17 died of cardiogenic shock in one week after surgery, the DFS and OS data of this patient were excluded.

DFS, disease‐free survival; OS, overall survival.

### 5.2 Next‐generation sequencing

The targeted capture pulldown and exon‐wide libraries were generated from native DNA using the xGen® Exome research panel (Integrated DNA Technologies, IL, USA) and the TruePrep DNA library prep lit V2 for Illumina (Vazyme, Nanjing, China). The paired‐end sequence data were generated using an Illumina NovaSeq 6000 machine (Illumina, San Diego, CA, USA).

### 5.3 Single nucleotide variants (SNV) calling from multi‐region WES

Pair‐end WES reads in FastQ format were aligned to the GRCh37 human reference genome using Burrows–Wheeler Aligner (BWA) v.7.17.^44^ The sequencing quality was measured by HsMetrics in Picard. Intermediate sorting and de‐duplication of Sam/Bam file was performed using sambamba v0.7.1. Recalibration of nonidentical reads were performed using bqsr. SNV and small INDEL were called by Strelka2 and Manta with default parameters.^45^ SNVs called by Strelka2 were further filtered by the following criteria: (1) sequencing depth ≥20 at variant sites in both control and tumour samples; (2) ≤5 alternative reads support the variant in germline sample; (3) ≥5 alternative reads support the variant in tumour sample Filtered variants were then annotated by Annovar and further filtered by the criteria; (4) <1% population frequency in all of the following database: Exome Aggregation Consortium (ExAC), ESP6500, the Genome Aggregation database (gnomAD). Resulting vcf files were converted to Maf format using vcf2maf for further analysis. TMB was defined as the total number of nonsynonymous somatic mutations per mega‐base (Mb) of the genome. It was derived from raw mutation count by dividing 38Mb which is the estimated exome size.^24^

### 5.4 Germline mutation calling

Germline mutation was called using best practices with the Genome Analysis Toolkit (GATK) HaplotypeCaller (version 3.6) as previously described (http://gatkforums.broadinstitute.org/gatk/discussion/1259/which‐training‐sets‐arguments‐should‐i‐use‐for‐running‐vqsr).^46^ Germline mutations are defined as alterations found in both tissue and blood DNA and labelled as germline by GATK.

### 5.5 Somatic copy number aberration analysis

CNV were called from aligned WES data using CNVkit.^47^ Tumour samples and matched normal pancreatic samples were analysed using CNVkit ‘batch’ command. Each input sample was median‐centred, followed by read‐depth bias corrections. The scatter and heatmap plots for CNV were generated. The GISTIC2.0 was used to identify regions of the genome that are significantly amplified or deleted across a set of samples.^48^ CNV burden was calculated based on the identified copy number variants as previously described,^49^ and then the average CNV burden was estimated for each patient. The wGII was calculated from the segments results from CNVkit.^50^

### 5.6 Driver mutation definition

Driver gene set was built by combining the two driver gene lists defined by previously studies.^51^, ^52^ Mutations on these driver genes were annotated with cosmic89_coding, oncoKB and other information by annovar. Candidate driver mutations included (i) nonsense, frame‐shift and splice‐site mutations and (ii) missense mutations, either with FATHMM‐MKL score >0.5 in the annotation of Catalogue of Somatic Mutation in Cancer (COSMIC) or identified as functional damaging by two or more functional analysis algorithms as follows: SIFT score = 0.0–0.05, Polyphen2‐classified ‘possibly damaging’ or ‘probably damaging’, MutationAssessor‐classified ‘medium’ or ‘high’ or FATHMM‐MKL predication score >0.5.

### 5.7 Clonal evolution analysis

Maf format mutation information was read and visualised using maftools.^53^ SCNA and CNV information for each sample was passed to PyClone to infer the subclonal clusters.^54^ The phylogenic evolution relationship among the subclones was constructed using revolver^55^ and sciClone.^56^ Trunk and branch variants were defined by their position in the evolutionary tree. Trunk mutations were defined as the mutations in the first ancestor clone of the lesions within individual tumours. The rest of the mutations were defined as branch mutations. To estimate the difference between trunk and branch mutations, filtered mutations were divided into trunk and branch categories. The signature of mutations in the two categories was extracted and compared with cosmic signatures using MutationalPatterns.^57^ To further validate the role of driver genes in cancer development, driver gene fold enrichment was determined between trunk and branch mutations as previously described.^58^ Briefly, enrichment odds ratio of mutated driver gene between trunk and branch categories^52^ was calculated by randomly sampling 12 out of 19 patients for their mutated genes. After 100 times of downsamplings, we tested the difference of odds ratio between two groups using Wilcoxon test.

### 5.8 Timing of mutations and copy number aberrations

Individual mutations were determined as clonal or subclonal by the cancer cell fraction (CCF) values from PyClone clustering results as previously described.^59^ Mutations with CCF values close to 1 were called clonal. Other mutations were considered subclonal. The clonal mutations were classified as early or late based on the mutation copy number calculated as previously described.^60^ A clonal mutation was called ‘clonal early’ if it was early across all regions or in the majority of regions. A clonal mutation was called ‘clonal late’ if it was late across all regions or in the majority of regions. The clonal mutations that could not be timed were called ‘clonal untimed’.^60^ For somatic copy number aberrations, chromosome arm gain or loss was defined and classified as clonal or subclonal as previously reported.^60^ In brief, copy number from facets results for segments in each sample was divided by the sample ploidy and log2 transformed. Gain and loss were defined with cutoff log2 (2.5/2) and log2 (1.5/2). Chromosomal arm gain or loss was called when >98% of chromosomal arm length show gain or loss in at least one sample of a patient. Arm gain or loss was classified as clonal when the same chromosomal arm shows >75% gain or loss across all the remaining samples from one patient. Otherwise, the chromosomal arm was defined as subclonal. Clonal chromosomal arm gain was further timed as early or late by the mutation copy number of all mutations on a given arm. Clonal chromosomal arm loss was further timed by the status of loss of heterozygosity in the chromosomal region.

### 5.9 RNA‐seq

Total RNA was isolated from tissue samples. mRNA was obtained from total RNA using oligo (dT) beads. The enriched mRNAs were fragmented and used as template to synthesise the first strand of cDNA. cDNA libraries were generated using Illumina TruSeq RNA Library Preparation Kit (Illumina) following the manufacturer's instructions. Sequencing was conducted using the Illumina NovaSeq 6000 system. Sequencing reads were qualified and trimmed for sequencing adaptors using Trimmomatic. Preprocessed reads were subsequently aligned to human genome (UCSC hg19 and corresponding annotation file) using STAR with default parameters.^61^ FPKM for each gene was calculated directly from aligned bam file using cufflinks software. Reads fell in each gene were counted by htseq‐count.^62^

### 5.10 Identification of differentially expressed genes

Identification of DEGs was conducted using GFOLD software.^63^ DEGs were defined as genes with an absolute GFOLD value greater than 2. The DEGs shared by all the lesions within the same tumour were defined as shared DEGs. Batch effects of FPKM values were removed using the combat function in R package sva.^64^ The fold‐change of gene expression in tumour samples compared with the corresponding control samples was used to visualise the expression difference of shared DEGs. Samples and genes were clustered using hierarchical clustering algorithm. PCA analysis was performed using R package factoextra. Pathway enrichment of shared DEGs was calculated using R package clusterProfiler with top 10 significantly enriched KEGG pathways (Benjamini adjusted *p* < .05).^65^

### 5.11 DNA methylation analysis and construction of phyloepigenetic trees

The DNA methylation profiles of 26 lesion samples, along with the matched normal tissue samples, from 7 randomly selected PDAC cases were obtained using MeDIP‐seq. Genomic DNA was isolated from the tissue samples using a QIAamp kit (Qiagen, Valencia, CA, USA) following the manufacturer's protocol. The quality and quantity of DNA were examined using a Nanodrop device (NanoDrop Technologies, Wilmington, DE, USA). After shearing the DNA using a Bioruptor (Diagenode, Liège, Belgium), end repair was performed using the AMPure XP Beads (Beckman Coulter, Indianapolis, IN, USA). A 3′ adenine overhang was added, followed by ligation with Illumina sequencing adapters and enrichment using PCR, as previously described.^66^ After cBot cluster generation, MeDIP‐seq was performed on an Illumina HiSeq 2500 device.

DMRs were identified using MACS2 approach^67^ and defined by false‐discovery rate (FDR)‐adjusted *p* values greater than .01. A private DMR referred to the DMR with the methylation peak only occurring in a single sample (*q* value < .01). A shared DMR referred to the DMR with methylation peaks appearing in multiple samples. Only private DMRs were used to construct the phyloepigenetic trees. For each tumour, enrichment fold‐based pairwise Euclidean distances were calculated using the complete set of private DMRs.

### 5.12 Tumour microenvironment (TME) analysis

TME was defined as the complicated ecosystem within bulk tumour tissue comprising of multicellular and stroma component such as immune cells (T and B lymphocytes, dendritic cells, natural killer cells, tumour‐associated macrophages, neutrophils and myeloid‐derived suppressor cells) and cancer‐associated fibroblasts. It is the primary site where tumour cells and the host immune cells interact and represents an additional source of intra‐tumoural heterogeneity.^68^ In this study, CIBERSORT was used to analyse the heterogeneity of the immune microenvironment in each tumour sample.^15^ Absolute immune cell abundance was calculated using a set of 22 immune cell reference profiles (LM22) on CIBERSORT website. It is an annotated gene signature matrix containing 547 marker genes that define 22 human immune cell subtypes such as T‐cell types, B‐cell types, plasma cells, natural killer (NK) cells and myeloid subpopulations.^15^ Tumour samples were clustered into two groups by their immune cell composition using R package ConsensusClusterPlus.^69^ The immune cell fraction in each sample was visualised with R package pheatmap.

### 5.13 Immunohistochemical analysis

Tumour tissue samples were subjected to IHC staining for markers of T follicular helper cells (CD4 and BCL6). The tumour tissue samples from PDAC patients were fixed with formalin and embedded in paraffin. Serial sections were prepared for haematoxylin‐eosin (HE) staining and IHC staining for CD4 and BCL6. And the following antibodies in accordance with the manufacturers’ recommendations: CD4 (PA0427, Leica Biosystems, Buffalo Grove, USA) and BCL6 (PA0204, Leica Biosystems, Buffalo Grove, USA). Labelled cells were incubated with horseradish peroxidase‐conjugated secondary antibody (DS9800, Leica Biosystems, Buffalo Grove, USA), followed by DAB staining using a DAB kit (Dako, Glostrup, Denmark). The proportion of positive cells in each tumour tissue sample was evaluated by two experienced pathologists using a semi‐quantitative scoring system. The density of T follicular helper cells was compared between two clusters according to RNA‐seq data.

### 5.14 Multiplex immunofluorescence analysis

Multiplex immunofluorescence (IF) staining for markers of T follicular helper cells (CD4 and BCL6) and tumour cells (Pan‐keratin) in tumour tissue samples was performed as described previously.^70^ The staining was obtained using PANO 7‐plex IHC kit (0004100100, Panovue, Beijing, China) and tyramide signal amplification (TSA) fluorescence kit (10021001050, Panovue, Beijing, China). The following primary antibodies were sequentially applied in accordance with the manufacturers’ recommendations: CD4 (ZM‐0418, Beijing Zhongshan Golden Bridge Biotechnology, Beijing, China), BCL6 (89369, Cell Signaling Technology, Massachusetts, USA) and Pan‐keratin (4545S, Cell Signaling Technology, Massachusetts, USA), followed by polymer horseradish peroxidase‐conjugated secondary antibody (10013001050, Panovue, Beijing, China) incubation. The nuclei staining was incubated by 4′‐6′ diamidino‐2‐phenylindole (DAPI, D9542, Sigma Aldrich).

The stained slides were scanned by Mantra System (PerkinElmer, Waltham, Massachusetts, USA). And the quantification of positively stained cells was performed using inform image analysis software (Version 2.4, PerkinElmer, Waltham, Massachusetts, USA).

### 5.15 Multi‐omics integration analysis

Gene‐level multi‐omics integration analysis was performed using CNAmet,^71^ a R package integrating copy number alteration, gene expression and DNA methylation information together. It calculates a gene wise weight score indicating genes alterations due to changes in DNA methylation and copy number levels. The correlation of *KDM6A* and *ZNF521* mRNA expression with the copy number alteration and methylation was assessed. The correlations of mRNA expression of *MDM4*, *RRAGC*, *HERC2*, *BIRC3* and *TSC2* with copy number alterations were evaluated.

On the pathway level, ActivePathways^16^ was also utilised to identify significantly altered pathways from multiple‐omics data. The input *p* values matrix was extracted from significant mutated genes (SMG), differentially expressed genes and differentially methylated genes. Significantly mutated genes were calculated by MutSigCV software on GenePattern Plateform. Differentially expressed genes between normal and tumour samples were calculated by DESeq2 R package. Differentially methylated genes between normal and tumour samples were calculated by MEDIPS R package. The pathway gmt files were downloaded from MsigDB database.

### 5.16 Statistical analysis

SPSS Statistics for Windows, version 22.0 (IBM Corp., Armonk, New York, USA) and ggpubr package^72^ in R^73^ were employed to analyse the correlations between clinical and biological variables. Where appropriate, the Fisher exact test or a non‐parametric test was used to compare categorical data. The differences in variables were compared using the log‐rank test. Spearman's correlation between gene expression and copy number was calculated using R or SPSS and visualised using the karyoploteR package in R.^74^ Clinical follow‐up evaluation was performed every 4 weeks, including physical examination, imaging and routine laboratory tests. Disease‐free survival (DFS) was defined as the time from surgery to locoregional recurrence or distant metastasis. Overall survival (OS) defined as the time from surgery to death from any cause or last follow‐up (censored patient). Patients who were still alive were censored on the last follow‐up date per chart review. The cut‐off date for analysis was May 31, 2021. The DFS and OS were calculated using the Kaplan–Meier method. A *p* value <.05 was considered statistically significant.

## CONFLICT OF INTEREST

The authors declare no potential conflicts of interest.

## Supporting information

Supporting Information

Supporting Information
