# Supplementary material for: Multi‐omics analysis of intra‐tumoural and inter‐tumoural heterogeneity in pancreatic ductal adenocarcinoma
Source: Clin Transl Med. 2022 Jan 21;12(1):e670. doi: 10.1002/ctm2.670 (PMC8782496; doi:10.1002/ctm2.670)

**Supplemental Figures**

**Supplementary Figure 1.** Microscopical morphology of the samples. Tumor heterogeneity was obvious within and across individual tumors. P, patient; L, lesion; N, matched adjacent nontumor tissue. Scale bar = 600μm.


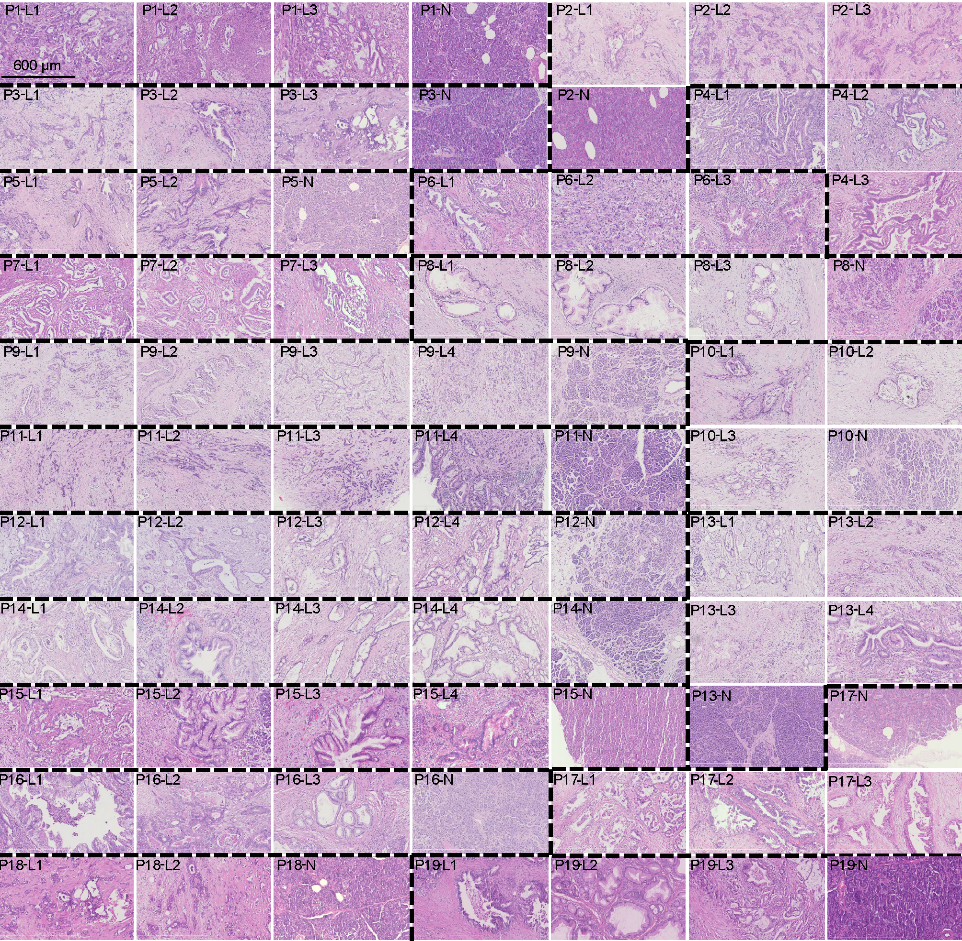


**Supplementary Figure 2.** Landscape of CNV reveals intratumor/intertumor heterogeneity of PDAC. (A) Distribution of total somatic CNVs of all lesions. Red denotes amplification, and blue indicates deletion. (B) The heatmap of CNVs. (C) Comparison of genome instability index (wGII) within and across individual tumors.

**Supplementary Figure 3**. Phylogenetic tree plotted by sciclone showed the clonal evolution of 4 patient.


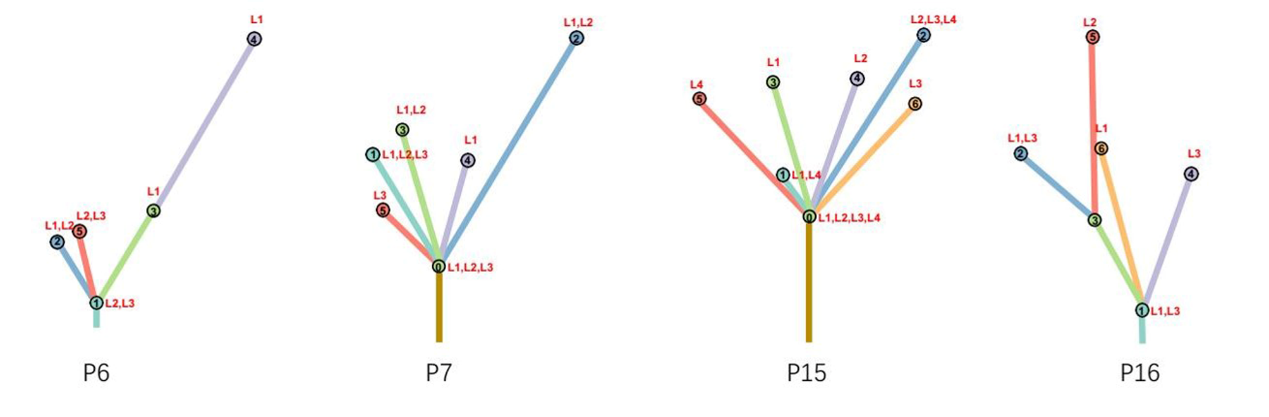


**Supplementary Figure 4.** (A) The distributions of DEGs across patients 8-19 using UpSet. (B)The distributions of DMRs across patients 8-14 using UpSet.


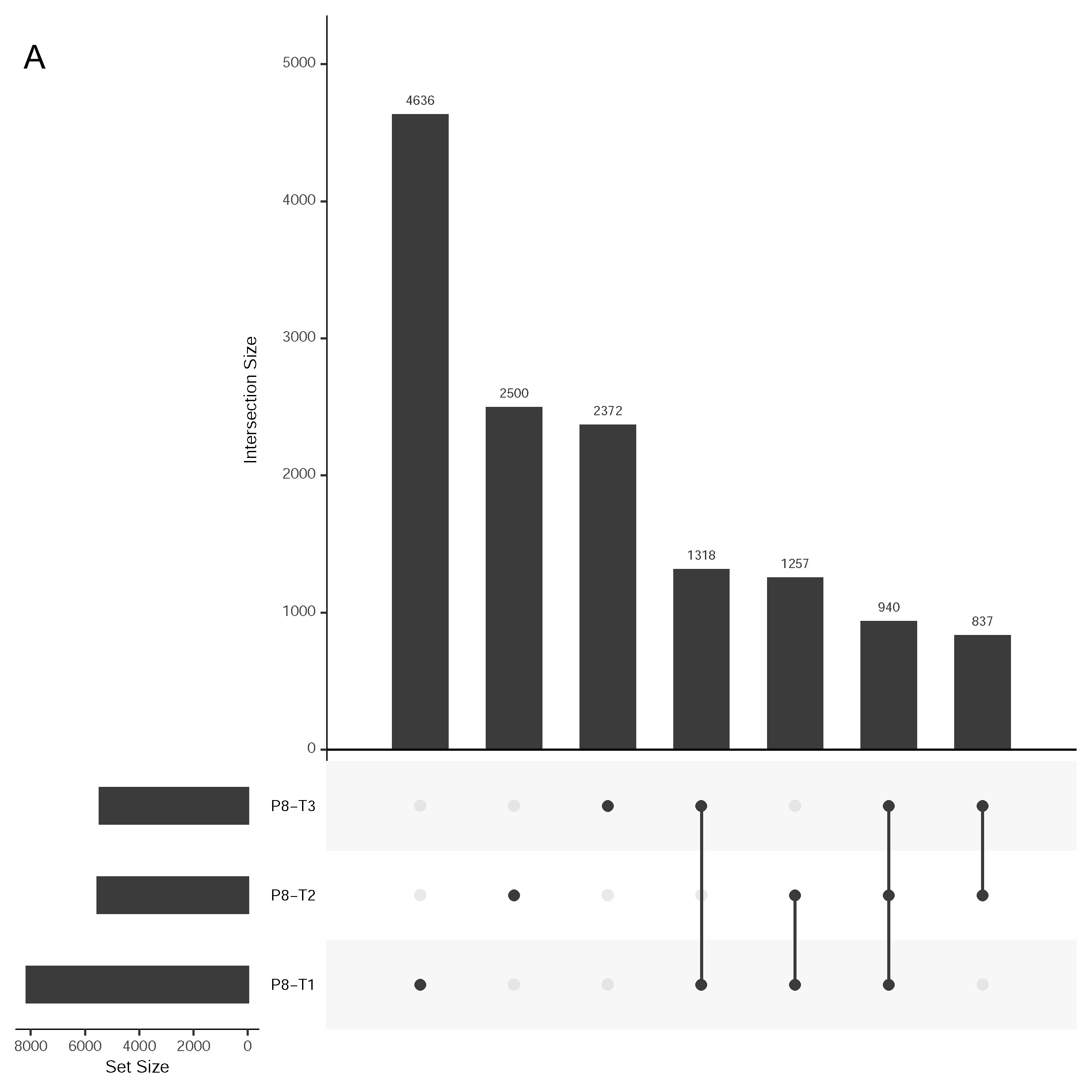

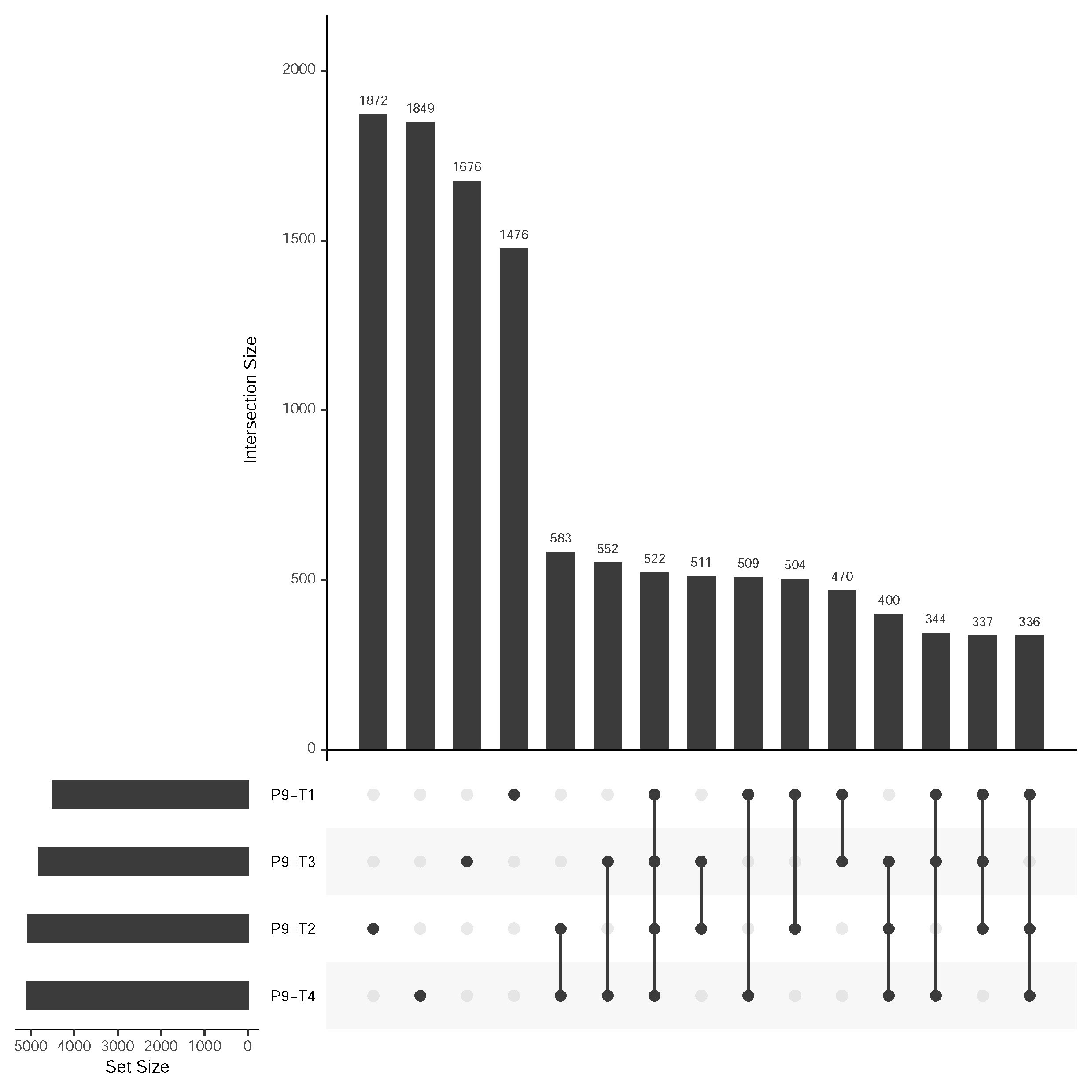

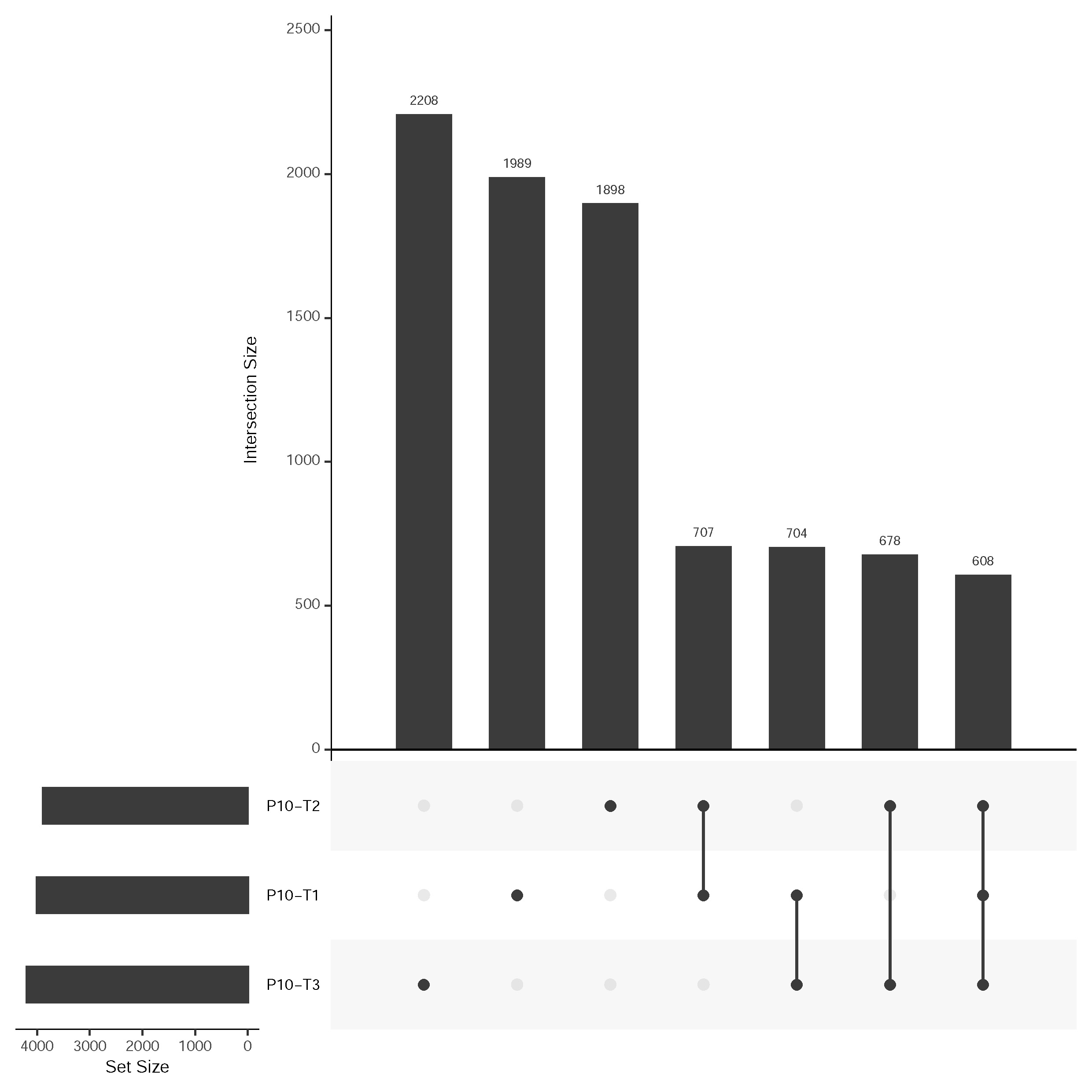

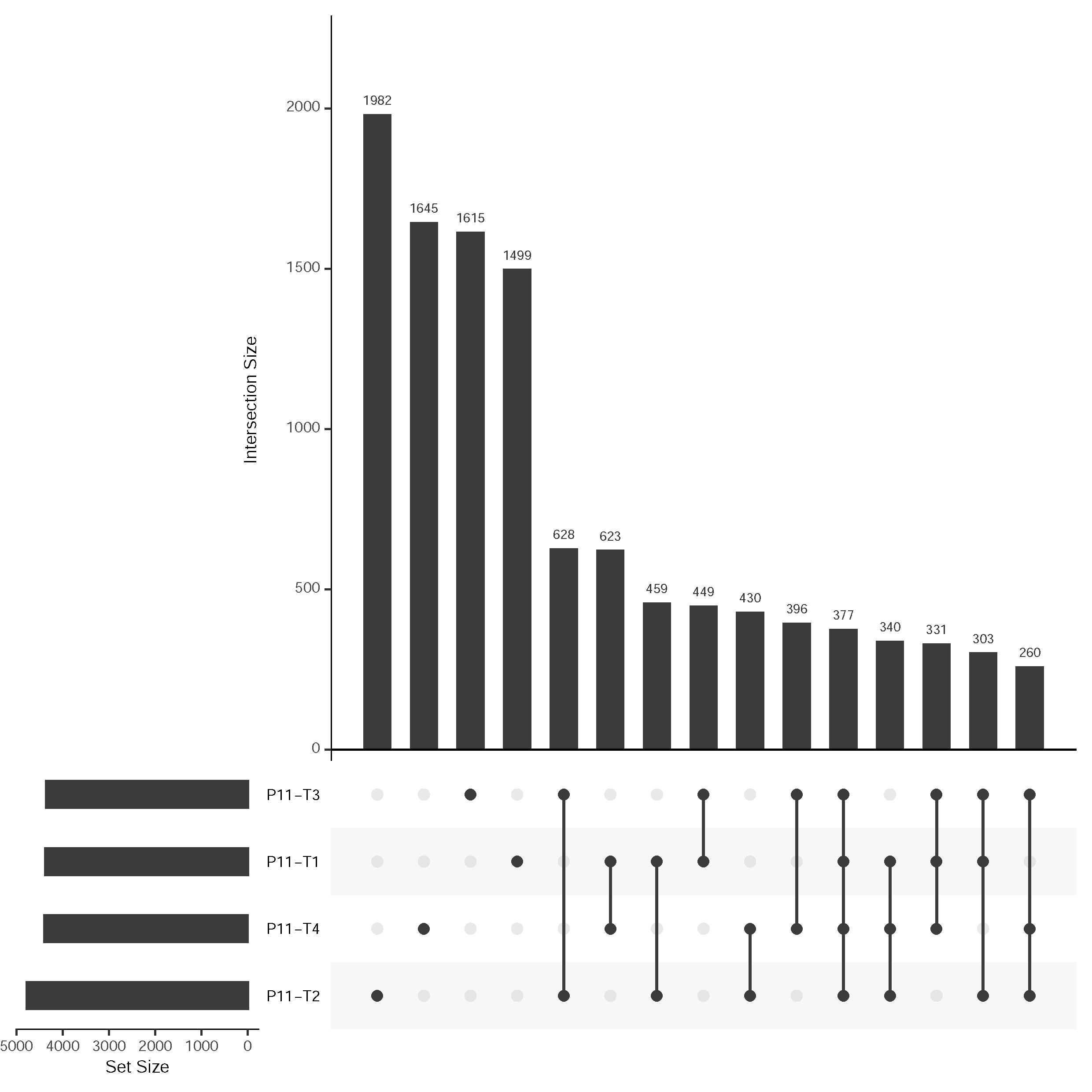

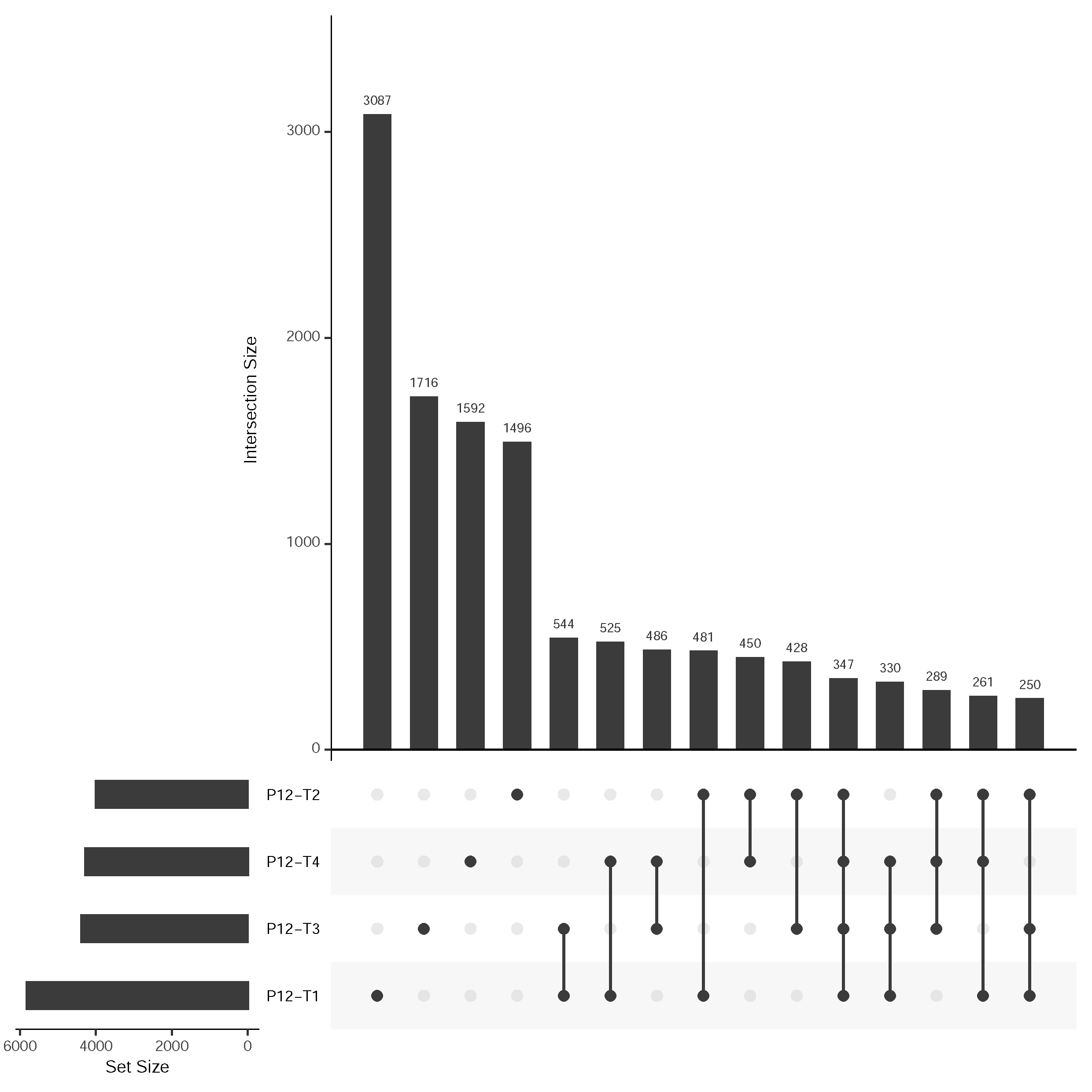

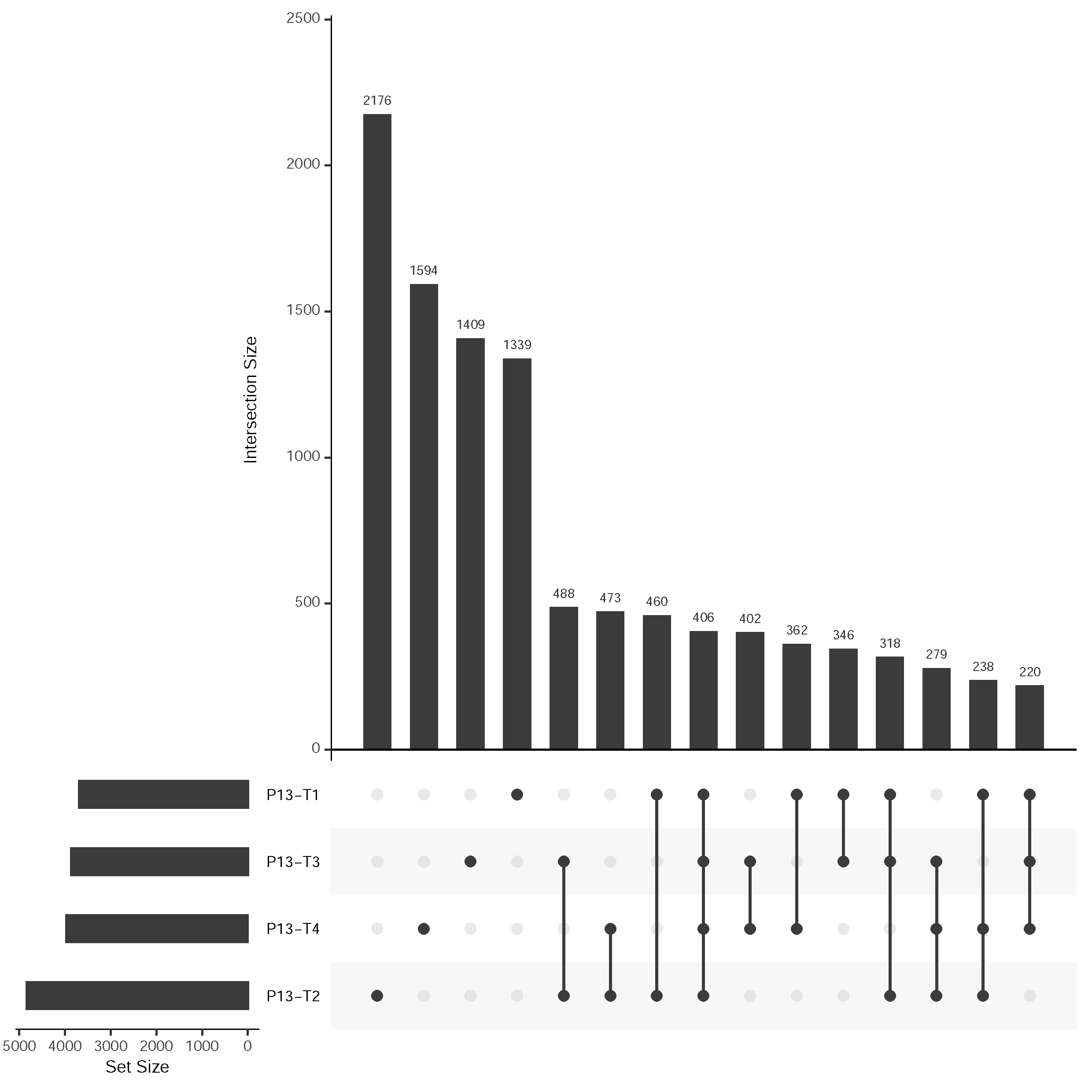

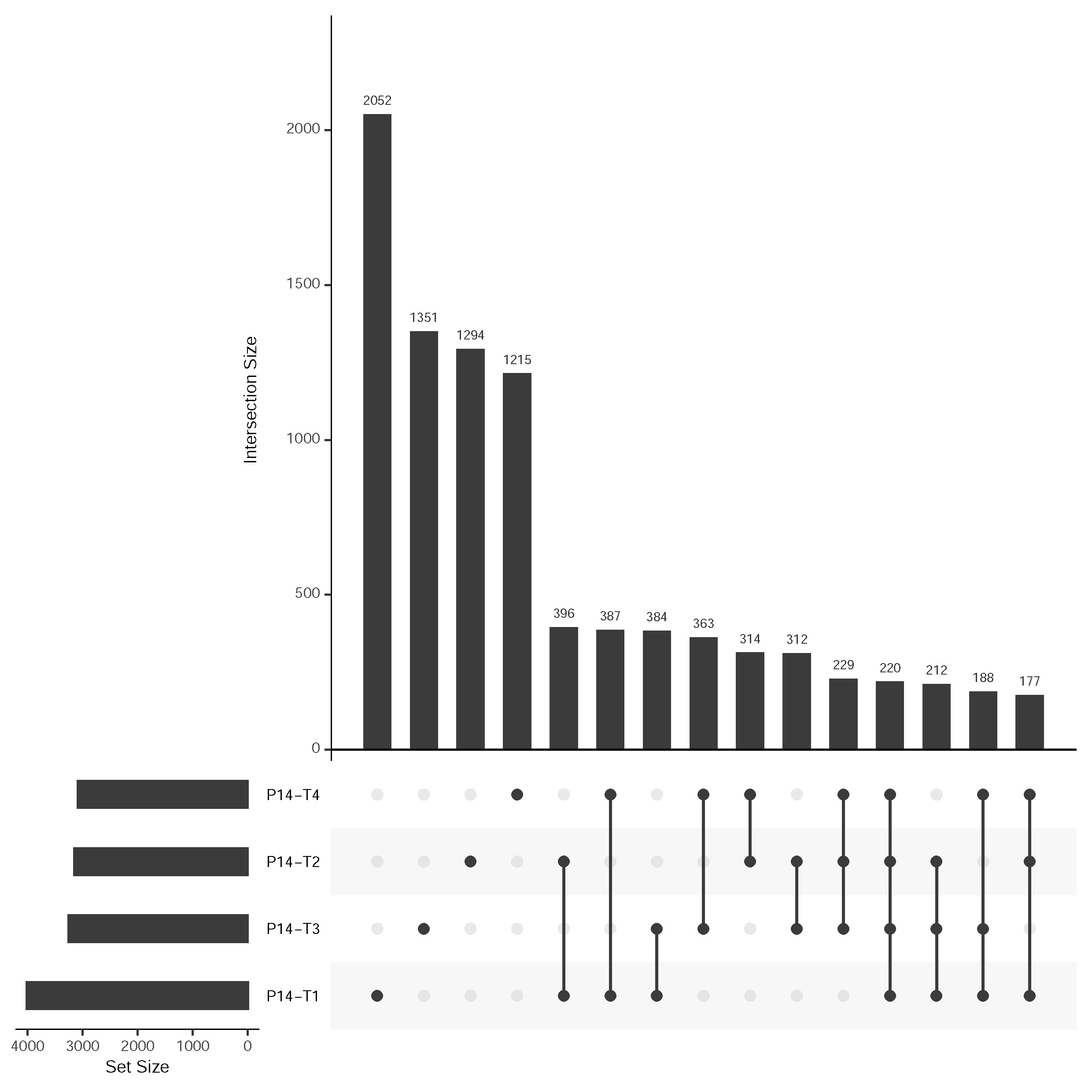

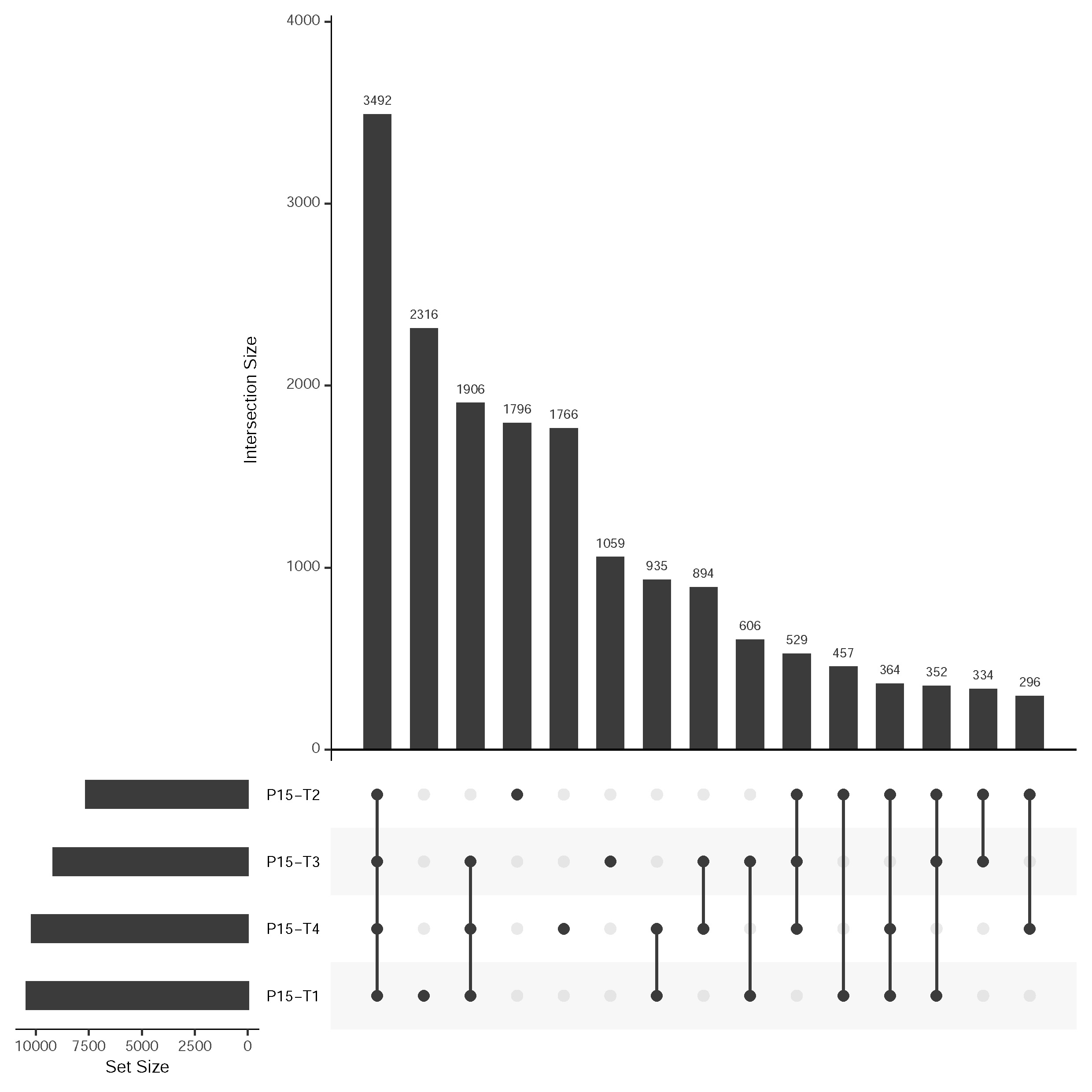

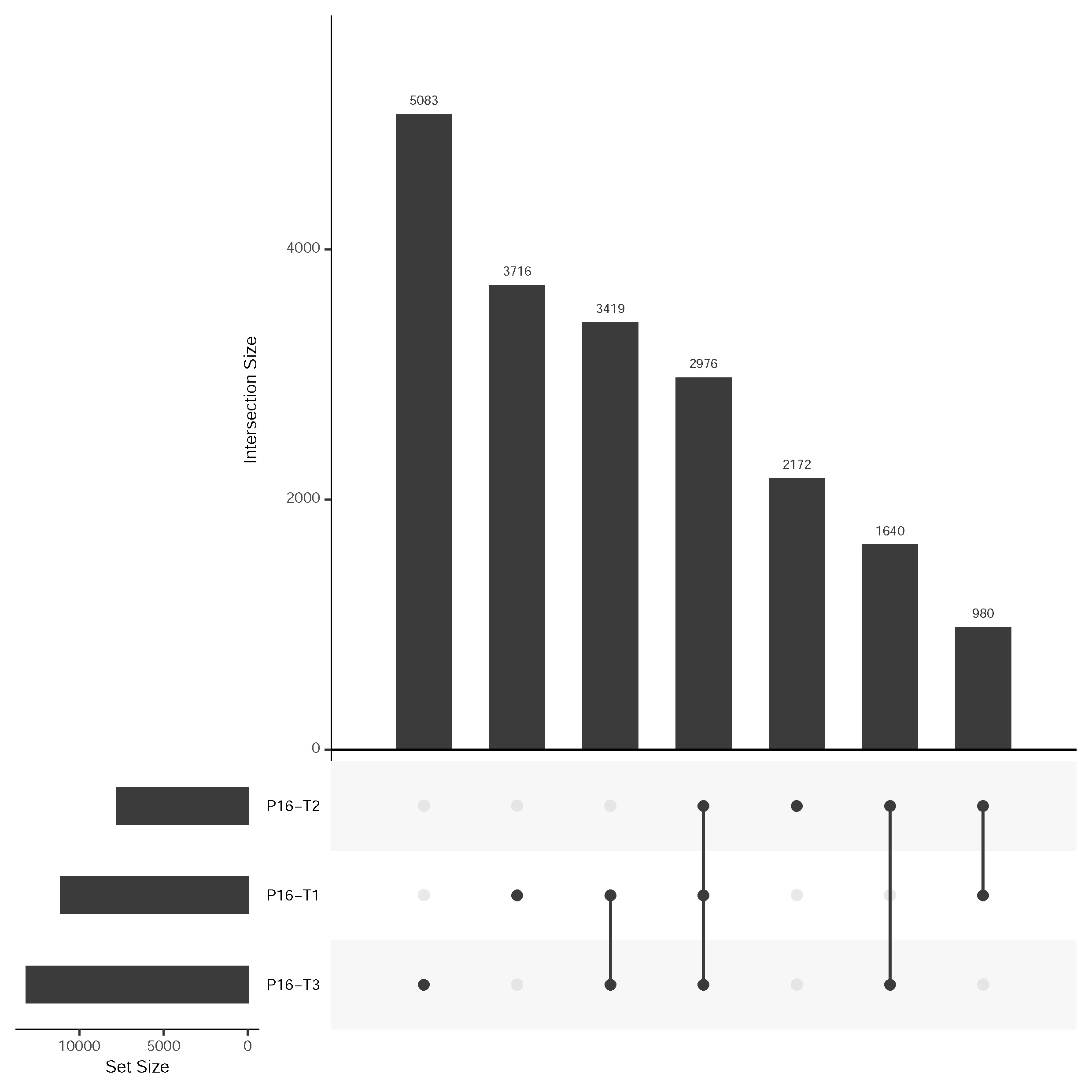

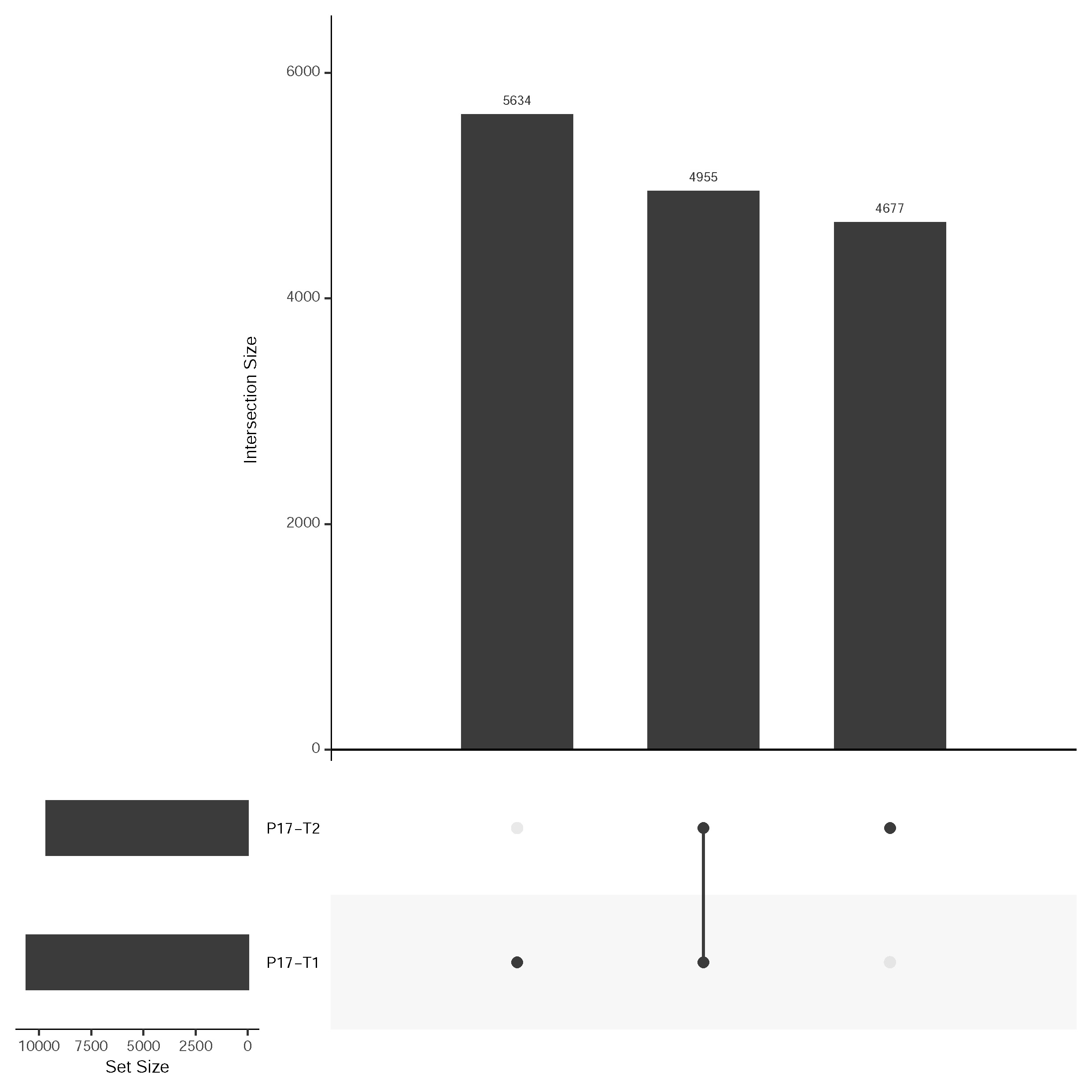

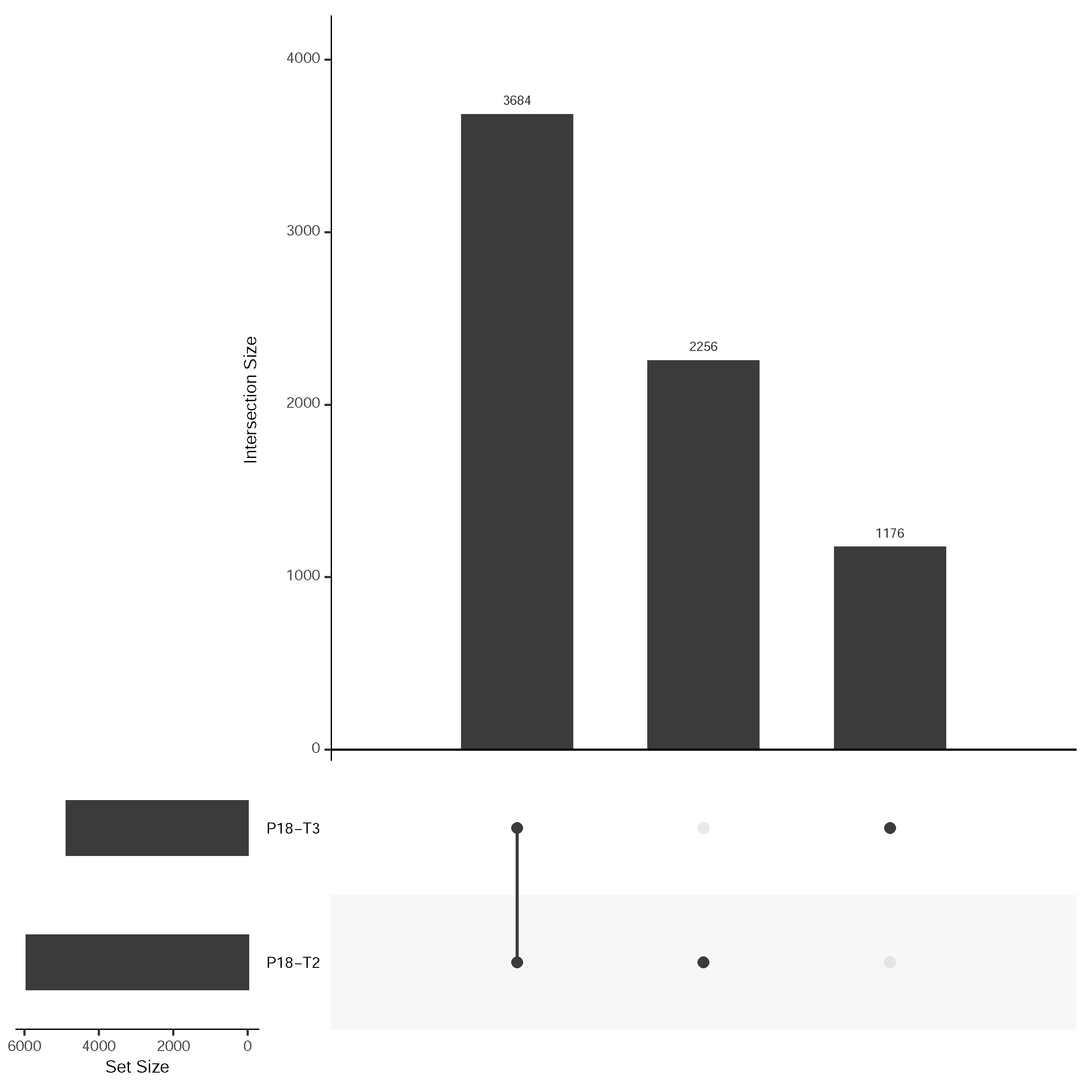

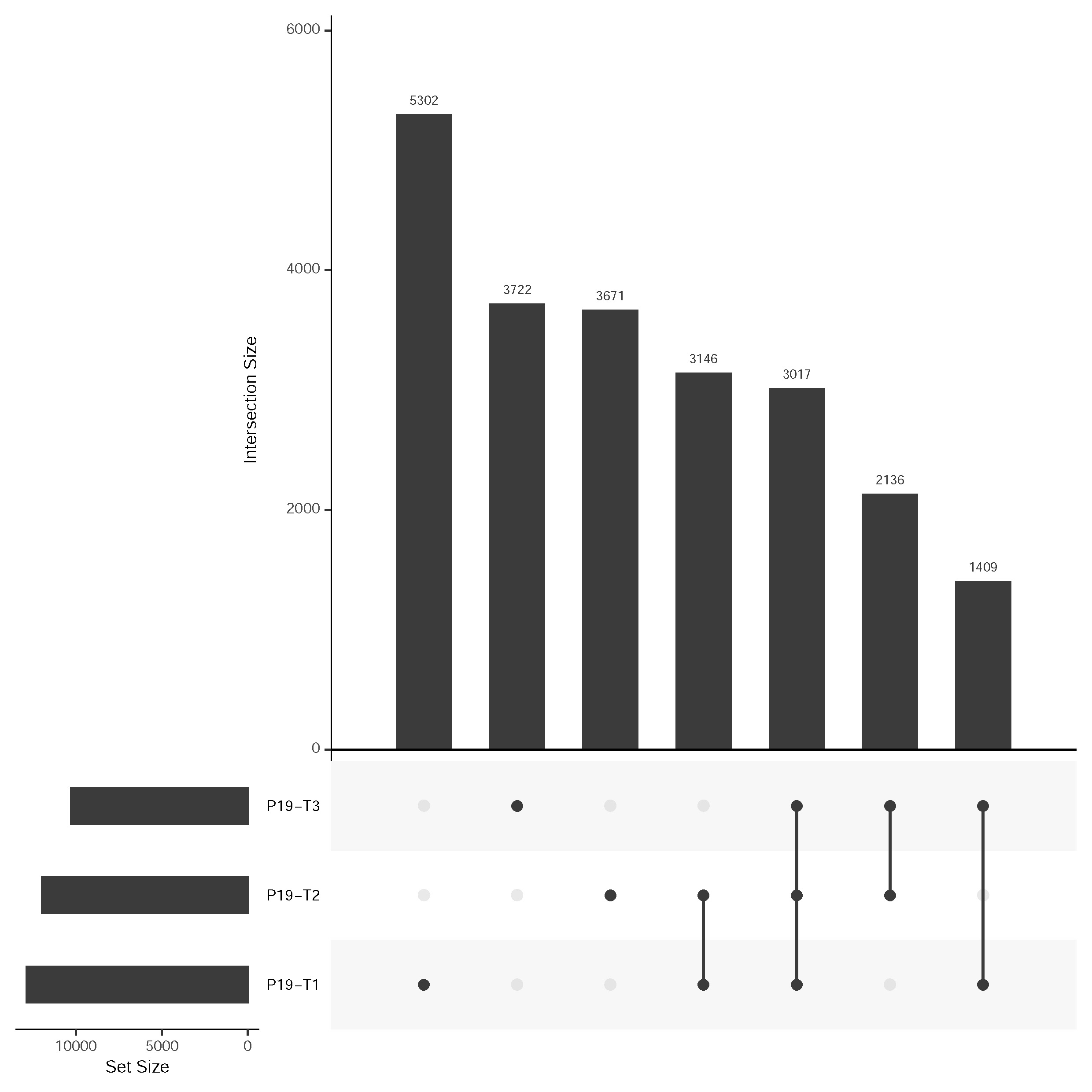

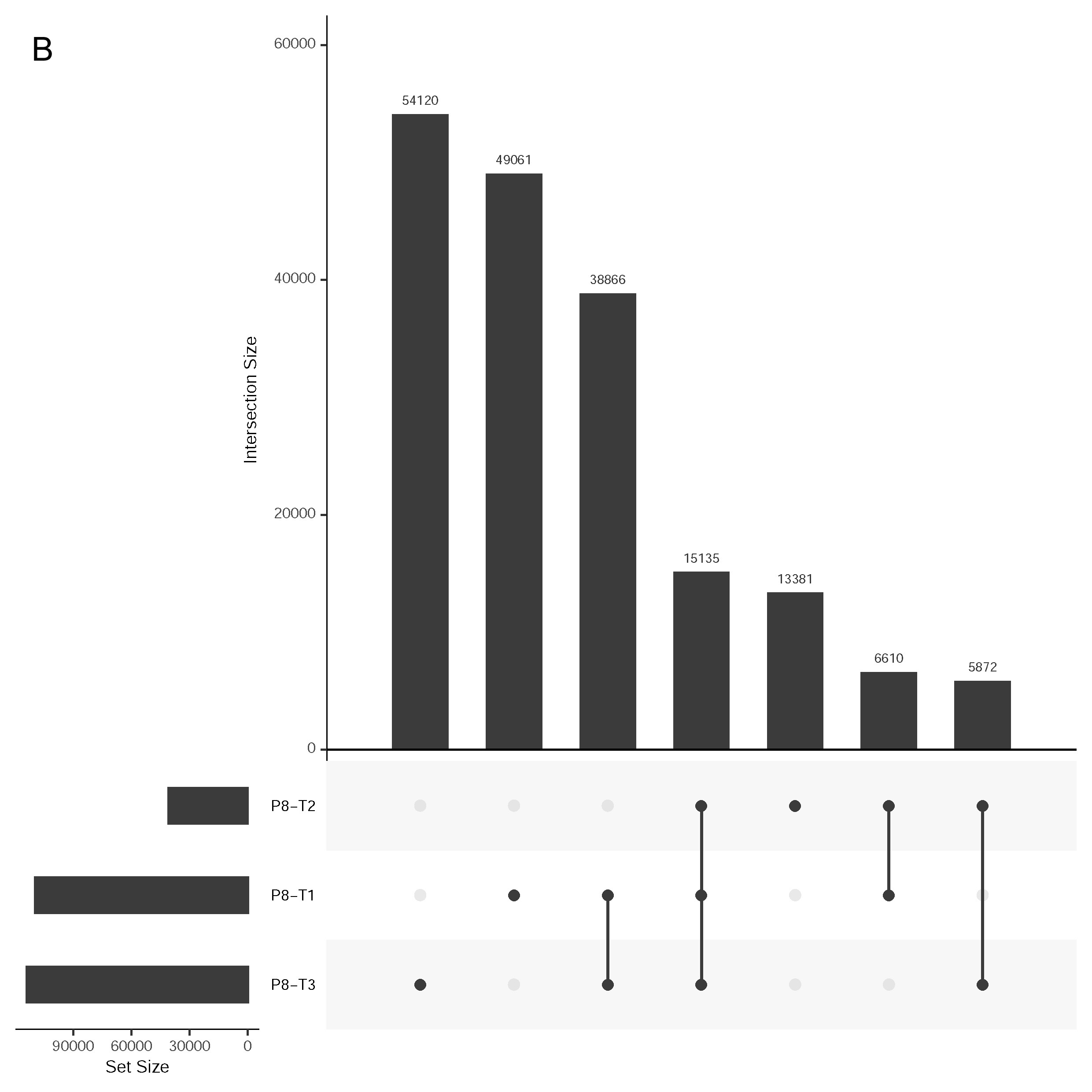

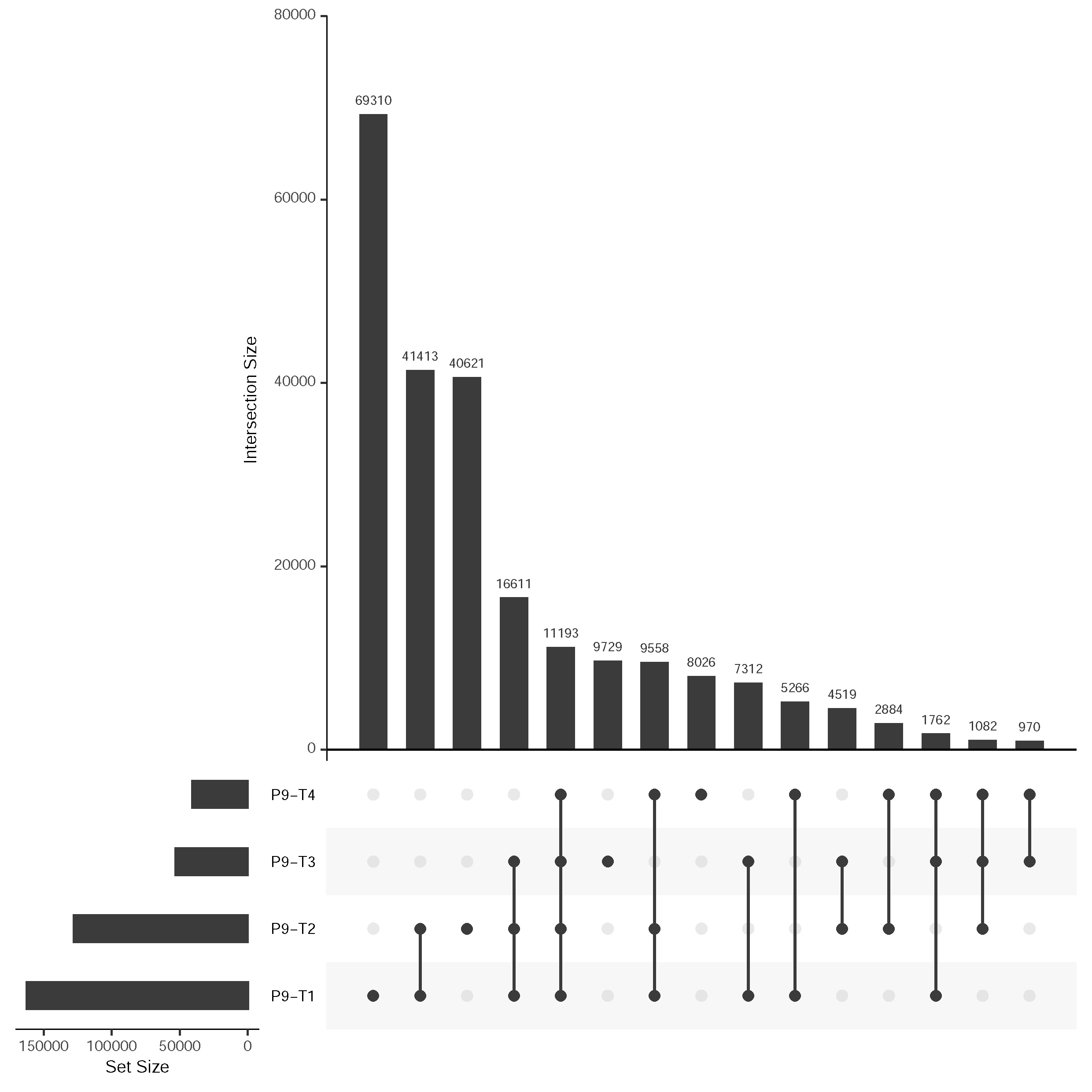

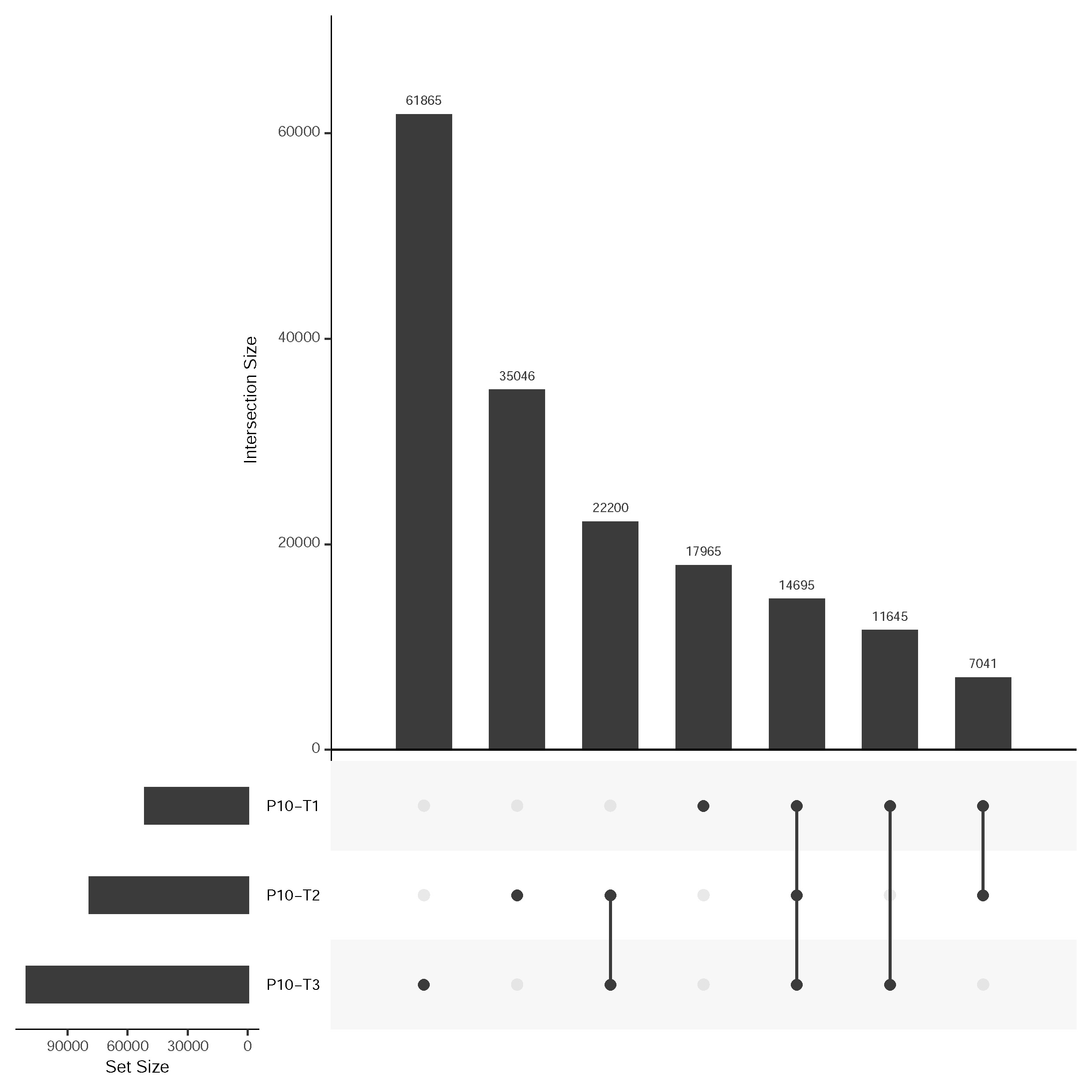

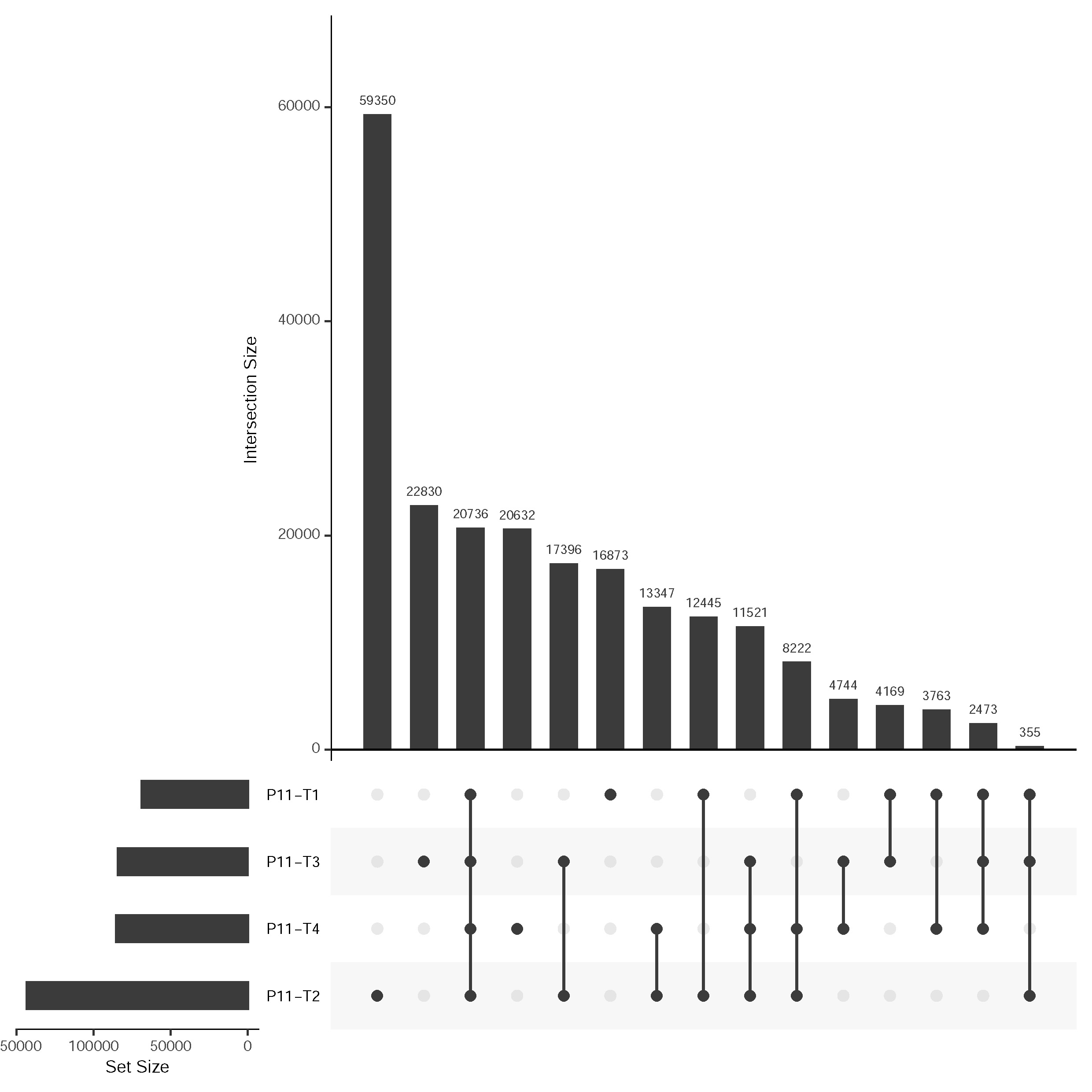

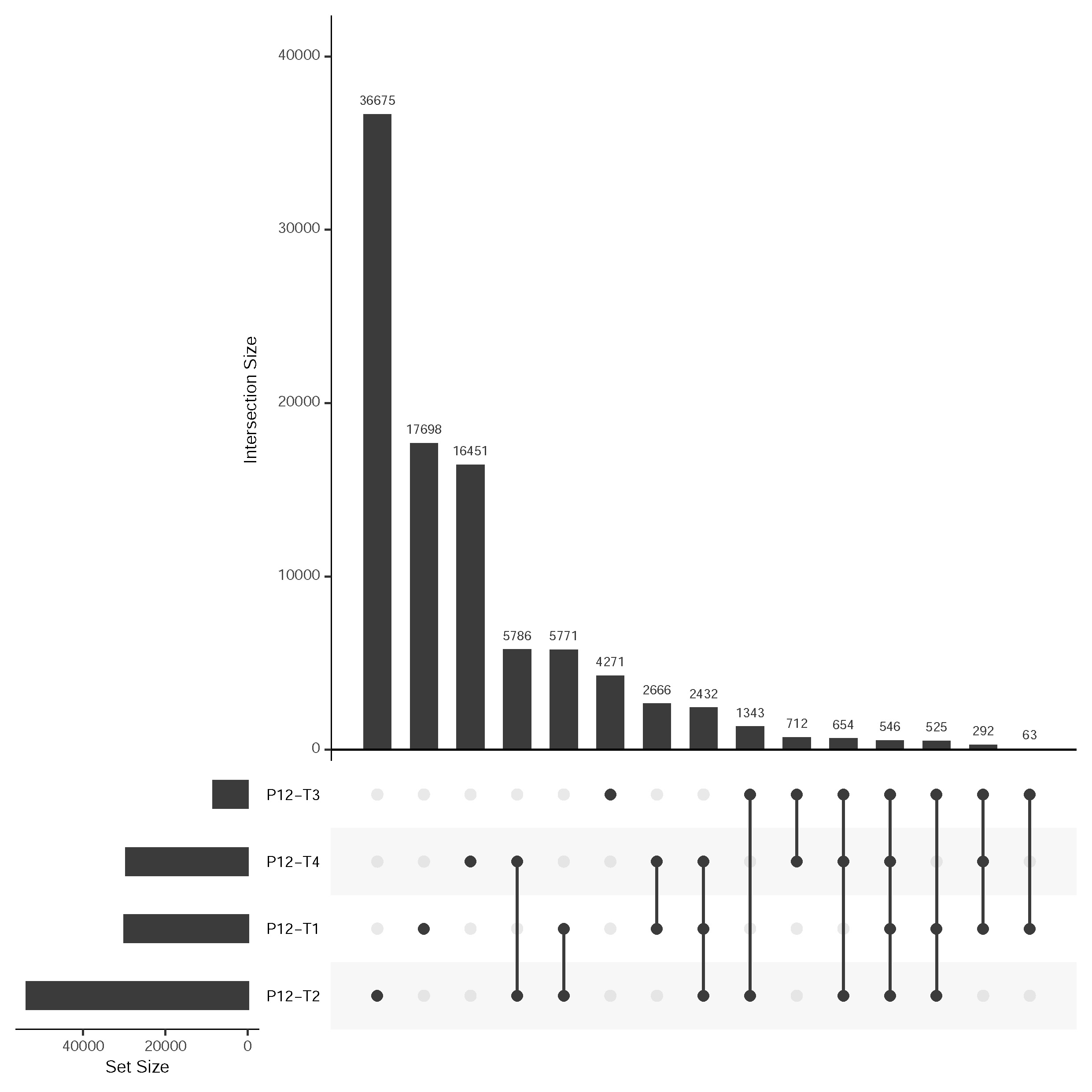

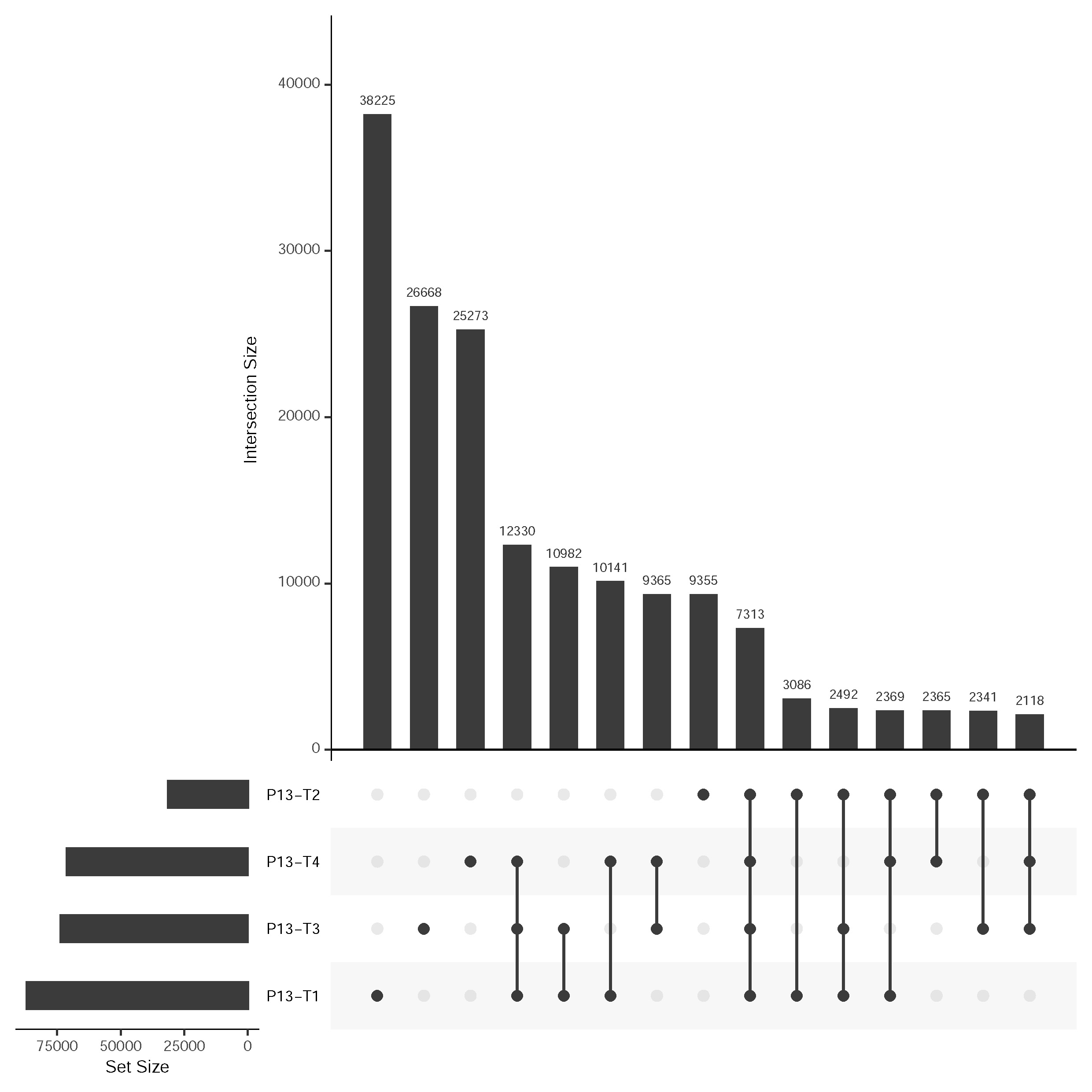

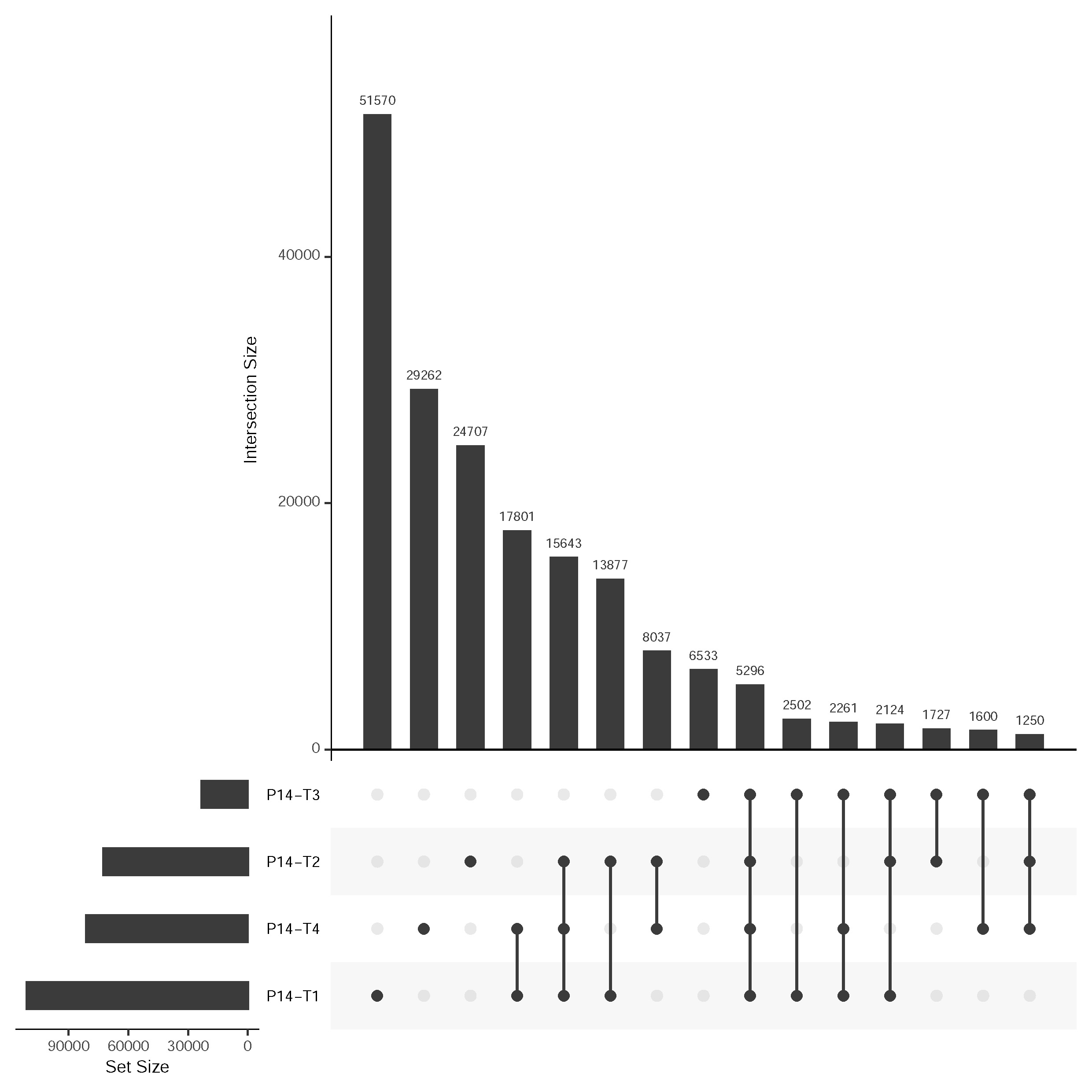


**Supplementary Figure 5.** (A) PCA cluster analysis of all samples using the fold change of gene expression, with PC1 accounting for 16.34% of the total variance and PC2 explaining 9.29%. (B) Immunohistochemical staining analysis of T follicular helper cells. ns, *p* > 0.05; *, *p* <= 0.05. (C&D) Multiplex immunofluorescence (IF) staining analysis of T follicular helper cells. ns, *p* > 0.05; **, *p* <= 0.01; ***, *p* <= 0.001.

**
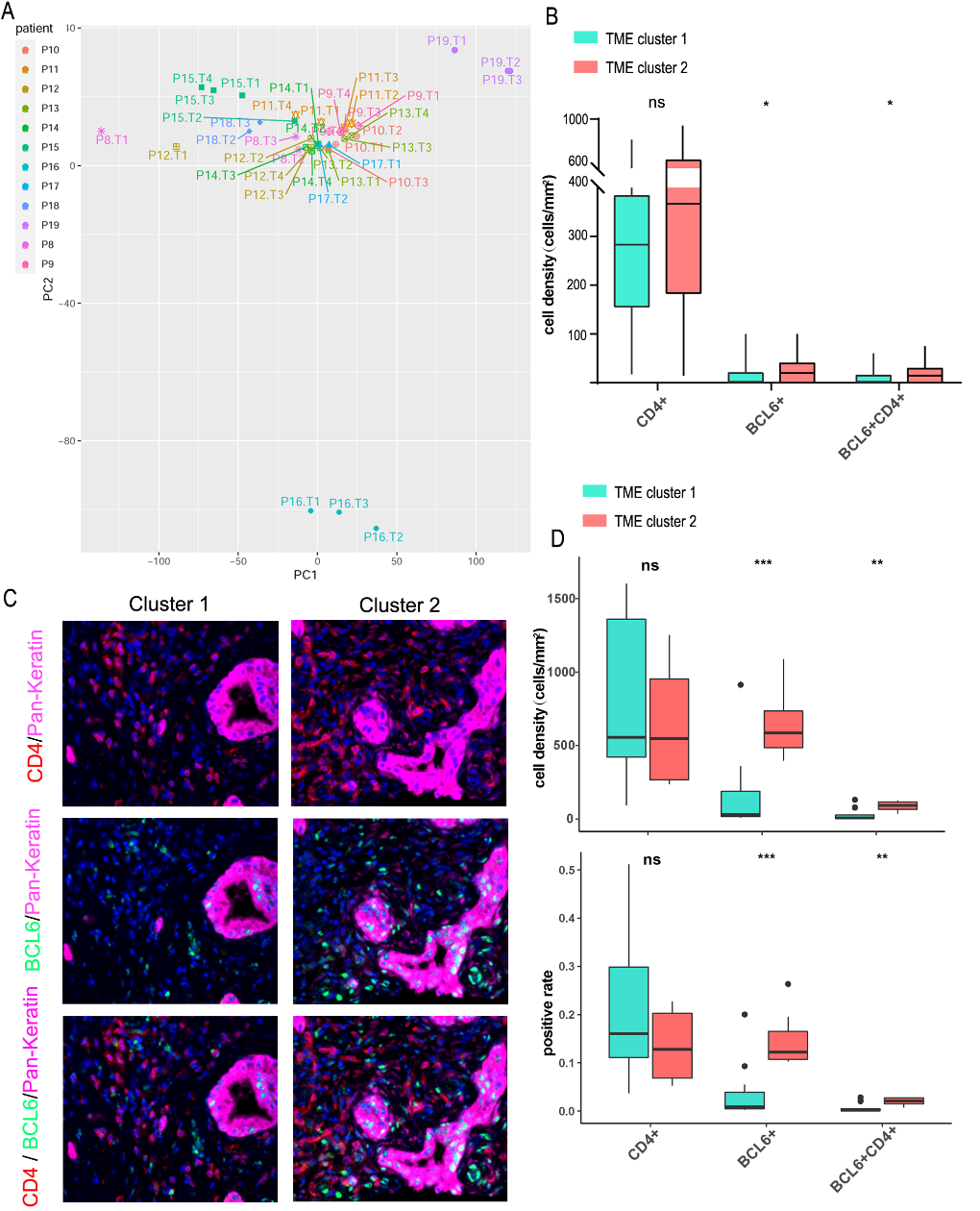
**

**Supplementary Figure 6.** (A) KEGG pathway enrichment of DEGs in four lesions of P15. (B) KEGG functional analysis of the genes with shared DMRs in P8, P9, P10, P13, P14.

**Supplementary Figure 7**. (A) Correlation between mRNA expression and CNV gain and loss of MDM4, RRAGC, HERC2, BIRC3, and TSC2. (B) The distribution of theirexpression, copy number, and methylation status across samples.


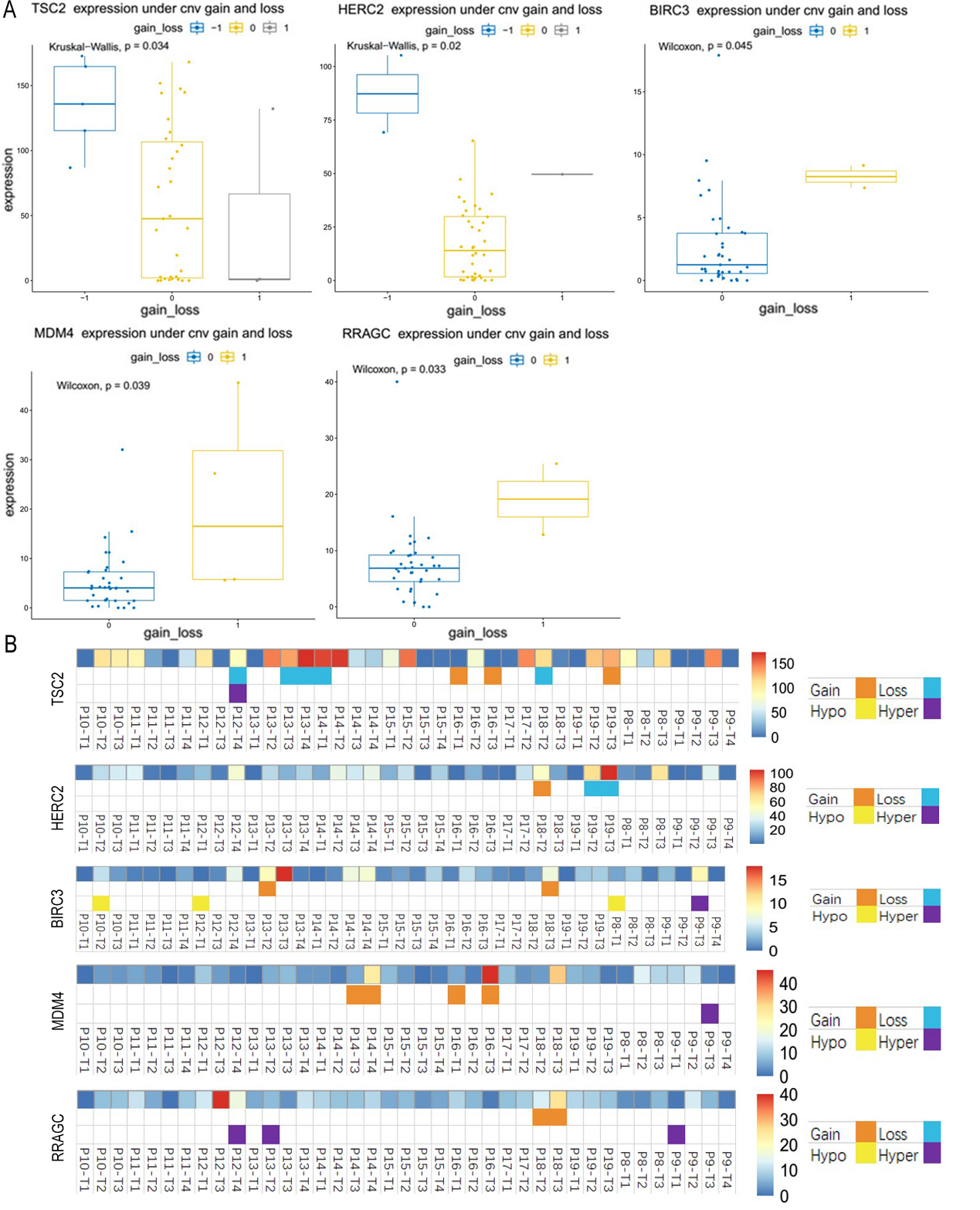


**Supplementary Figure 8.** Integrative pathway analysis of multi-omics data for KEGG pathway enrichment (A) and GO biological processes enrichment (B).

**
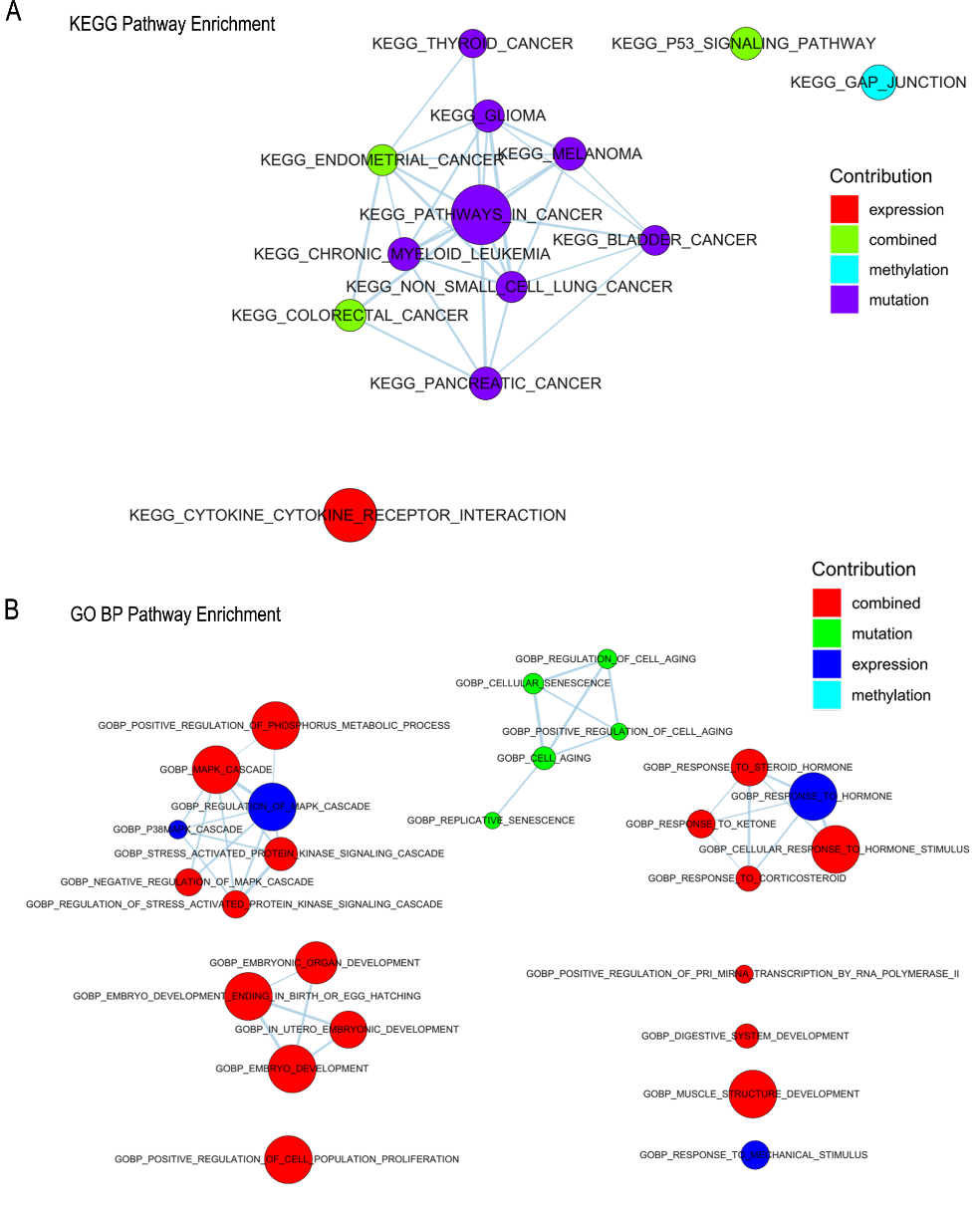
**

**Supplementary Figure 9.** Correlation of ZNF521 (A) or KDM6A (B) expression with disease-free survival and overall survival of patients in this study.


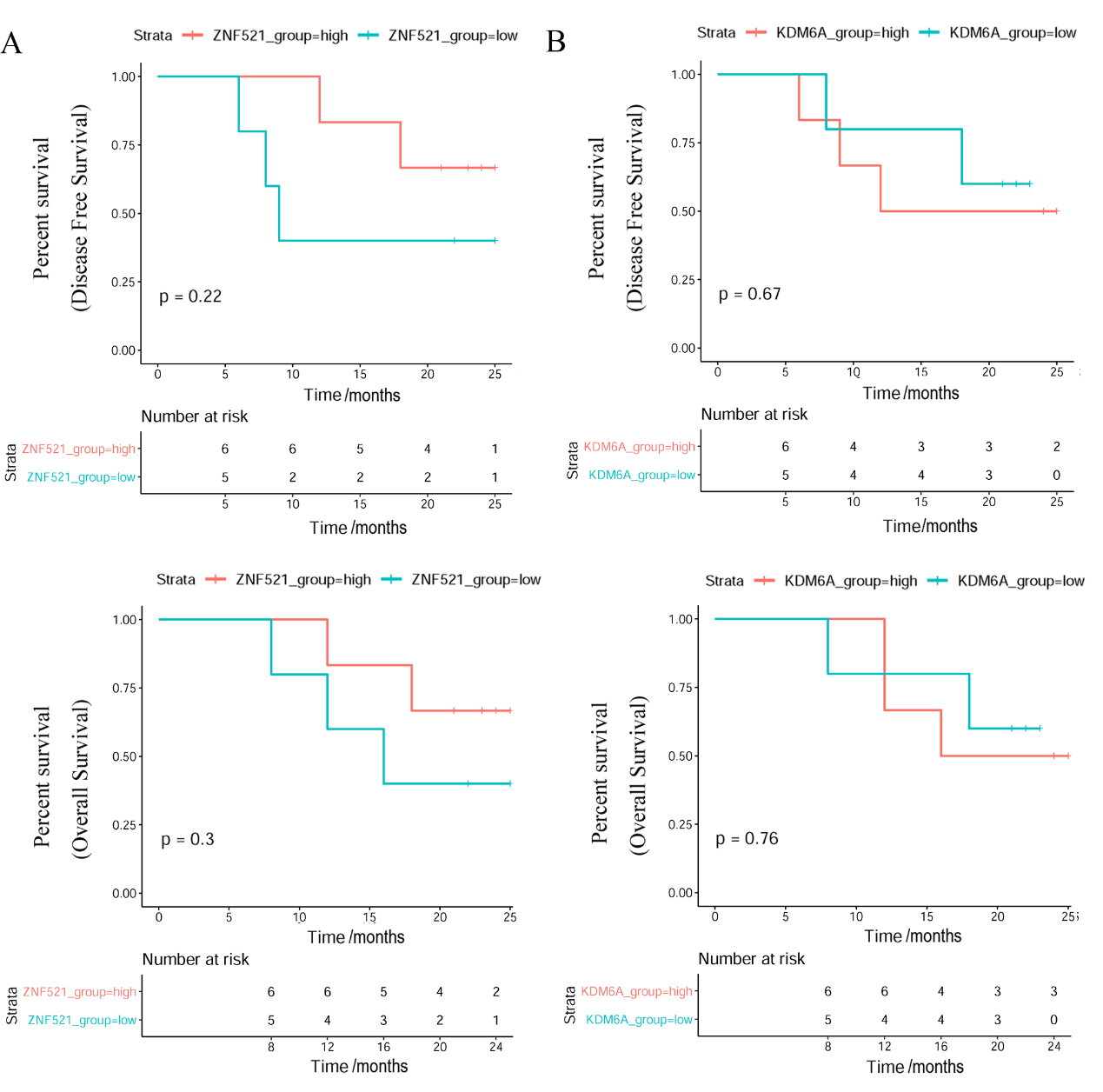

Supplement: Supplementary file 1 — Supporting Information [file CTM2-12-e670-s002.docx]
